# Revealing the mechanisms of the bioactive ingredients accumulation in *Polygonatum cyrtonema* by multiomics analyses

**DOI:** 10.3389/fpls.2022.1055721

**Published:** 2022-11-16

**Authors:** Ting Xue, Miaohua Zhao, Jing Chen, Youqiang Chen, Chuanhai Zhang, Baoyin Li

**Affiliations:** ^1^ Fujian Provincial Key Laboratory for Plant Eco-physiology, State Key Laboratory for Subtropical Mountain Ecology of the Ministry of Science and Technology and Fujian Province, College of Geographical Sciences, Fujian Normal University, Fuzhou, China; ^2^ College of Life Sciences, Fujian Normal University, Fuzhou, China; ^3^ Fujian Provincial Key Laboratory of Eco-Industrial Green Technology, College of Ecology and Resource Engineering, Wuyi University, Nanping, China

**Keywords:** *Polygonatum cyrtonema*, metabolome, mRNA, miRNA, WGCNA, bioactive ingredients

## Abstract

*Polygonatum cyrtonema* is a medicinal and edible herb rich in polysaccharides, steroidal saponins, and flavonoids that has been widely used as a food, vegetable, and medicine over the years. Although previous studies have preliminarily explored the metabolic and transcriptional regulatory mechanisms of the main secondary metabolites in *P. cyrtonema*, the complex mechanism of microRNA (miRNA)-mediated posttranscriptional regulation remains unclear. Metabolome analysis showed that iso-ophiopogonanone B, (25S)-pratioside D1, disporopsin, and isodiosgenin-Glc-Glc, which are associated with intermediates in the flavonoids and saponins pathways, were significantly upregulated in the stem and leaf compared with the rhizome, and most saccharides, including arabinose, cellobiose, maltotetraose, and panose, showed the opposite trend, suggesting that they may contribute to the formation and accumulation of the main active ingredients in *P. cyrtonema*. We found that 4-hydroxymandelonitrile have a relatively good inhibitory effect on α-glucosidase, indicating that it may play a role in hypoglycemic functions. Transcriptome and weighted gene coexpression network analysis (WGCNA) were combined to reveal several candidate genes involved in the accumulation of polysaccharides, saponins, and flavonoids, including *PcSQLE*, *PcCYP71A1*, *PcSUS*, *PcFK*, and *PcMYB102*. Integrated analyses of miRNAs and messengerRNAs (mRNAs) showed that novel_miR14, novel_miR49, novel_miR75, and aof_miR164 were negatively correlated with alpha-linolenic acid metabolism and the mitogen activated protein kinase (MAPK) signaling pathway, including *PcAOS*, *PcSPLA2*, *PcFRK1*, and *PcDELLA*, indicating that these miRNAs may coordinately regulate the biosynthesis of other secondary metabolites in *P. cyrtonema*. These findings will facilitate in-depth research on the functions of these miRNAs and mRNAs related to the main active substances for pathological and biological regulation, which will be beneficial to provide theoretical guidance for the molecular breeding of *P. cyrtonema*.

## Highlights

• The intermediates of flavonoids and saponins pathways were significantly up-regulated in the stem and leaf compared with rhizome.

• *PcFK, PcF3H, PcAMY, PcCYP71A1,* and *PcSUS* exhibited a significantly high expression level and were associated with secondary metabolites accumulation. 

•*PcMYB3, PcMYB97, PcMYB102, PcMYB33,* and *PcMYB61* are correlated with flavonoids content.

• A total of 169 miRNAs and 3,432 target genes were identified.

• aof_miR164 was negatively correlated with *PcAOS, PcSPLA2, PcFRK1,* and *PcDELLA*.

## Introduction

*Polygonatum cyrtonema* is a kind of medicinal and edible herb belonging to the genus *Polygonatum* (comprising ~70 species) within the family Liliaceae, which was incorporated into the Chinese Pharmacopoeia by 2020 together with *P. sibiricum*, *P. kingianum*, and *P. odoratum* (Zhao et al., 2018; Zhang et al., 2020; Zhang et al., 2021; Zhang et al., 2022). The rhizome of *P. cyrtonema* has the functions of moistening the lung, replenishing qi, invigorating the spleen, nourishing yin, and reinforcing the kidney and has been widely applied to treat various diseases for more than 2000 years in Asia (Liu et al., 2021; Liao and Wu, 2021). Modern pharmacological studies have shown that the rhizome of *P. cyrtonema* is rich in polysaccharides, steroidal saponins, flavonoids, and alkaloids, which have multiple biological activities, including antimicrobial, regulating blood lipids, antiaging, lowering blood sugar, and anti-inflammatory activity (Zhao and Li, 2015; Ma et al., 2019; Liao et al., 2020; Wu et al., 2022). With its extremely high economic value and social benefit, the development and application of *P. cyrtonema* have broad market prospects in the fields of medicine, food, and health products.

Numerous studies have focused on resource investigation, reproductive characteristics, chemical constituents, seed germination, ecological suitability, pharmacological effects, breeding and cultivation of *P. cyrtonema* (Zhao and Li, 2015; Xu et al., 2016; Zhang et al., 2020; Li et al., 2021). Zhang et al. reported that the main factor affecting the growth of *P. cyrtonema* was the monthly precipitation based on MaxEnt and the distribution range of literature records (Zhang et al., 2021). Most of the seed germination inhibitors from *P. cyrtonema* could inhibit root growth, chlorophyll synthesis and water absorption, and a GA3 concentration of 0.3 mg/L exerted an antagonistic effect on the germination inhibitor (Liu et al., 2021). The polysaccharides extracted from *P. cyrtonema* had good hypoglycemic and antiosteoporosis effects on a model of *juvenile zebrafish* with type 2 diabetes and osteoporosis (Fan et al., 2020). However, the above studies have little involvement in the molecular mechanism of the main bioactive ingredients (such as polysaccharides, flavonoids, and saponins) biosynthesis in *P. cyrtonema*. Understanding the genetic basis of *P. cyrtonema* can also help to harbor useful molecular information that can underlie secondary metabolite biosynthesis. Flow cytometry analysis found that *P. kingianum*, *P. sibiricum*, *P. odoratum*, *P. multiflorum*, and *P. cyrtonema* belong to the genus *Polygonatum* with complex ploidy and a large genome size (from 11 to 14 Gb), suggesting that the whole genome sequencing of *P. cyrtonema* is a huge challenge (Zhang et al., 2022). High-throughput transcriptional sequencing has been widely used in the biosynthetic pathway of secondary metabolites in various plants due to its low sequencing cost, large amount of data, and high accuracy. Transcriptome sequencing was used to identify candidate genes associated with polysaccharides accumulation in *P. cyrtonema* (Wang et al., 2019). Integrated transcriptome and metabolome analysis found that germinated seeds could inhibit lignin synthesis, which is beneficial to the germination of *P. cyrtonema* seeds (Liu et al., 2021). Transcriptome analysis of the rhizome of *P. cyrtonema* identified 27 encoded enzymes associated with steroidal saponins biosynthesis (Liao et al., 2020). Transcriptome sequencing of four tissues (leaf, stem, rhizome, and root) in *P. cyrtonema* revealed several transcripts involved in the synthesis of polysaccharides and saponins and suggested a possible biosynthesis pathway (Zhu et al., 2020). Although the above studies have preliminarily explored the metabolic and transcriptional regulatory mechanisms of the main secondary metabolites in *P. cyrtonema*, the complex mechanism of miRNA-mediated posttranscriptional regulation remains unclear.

It is of great significance to elucidate the miRNAs associated with main secondary metabolite biosynthesis and their regulatory mechanisms in *P. cyrtonema* for cultivating high-quality varieties. miRNA is an important regulatory factor of plant gene expression and is involved in signal transduction, stress, and secondary metabolism (Niu et al., 2016; Qiao et al., 2017; Yang et al., 2021; Li et al., 2022). miR156, miR159/319, miR165/166, miR778, and miR828/858 have different regulatory mechanisms for secondary metabolite biosynthesis (Jeyaraj et al., 2017; Zheng et al., 2021; Xue et al., 2022; Zheng et al., 2022). In this study, we first performed mRNA and miRNA sequencing of roots, rhizomes, stems, and leaves in *P. cyrtonema* to screen common and specific differentially expressed genes (DEGs) and miRNAs (DEMs), analyzing their potential functions in secondary metabolite biosynthesis. Additionally, a widely targeted metabolomic analysis was used to detect the changes in secondary metabolites in different tissues of *P. cyrtonema*. mRNA-miRNA coexpression and weighted gene coexpression network analyses were combined to mine the candidate genes or miRNAs associated with the accumulation and regulation of secondary metabolites, especially polysaccharides, saponins, and flavonoids. These findings will facilitate a deeper understanding of the molecular mechanisms of secondary metabolite biosynthesis at the omics level.

## Materials and methods

### Plant materials

**Figure 1:**
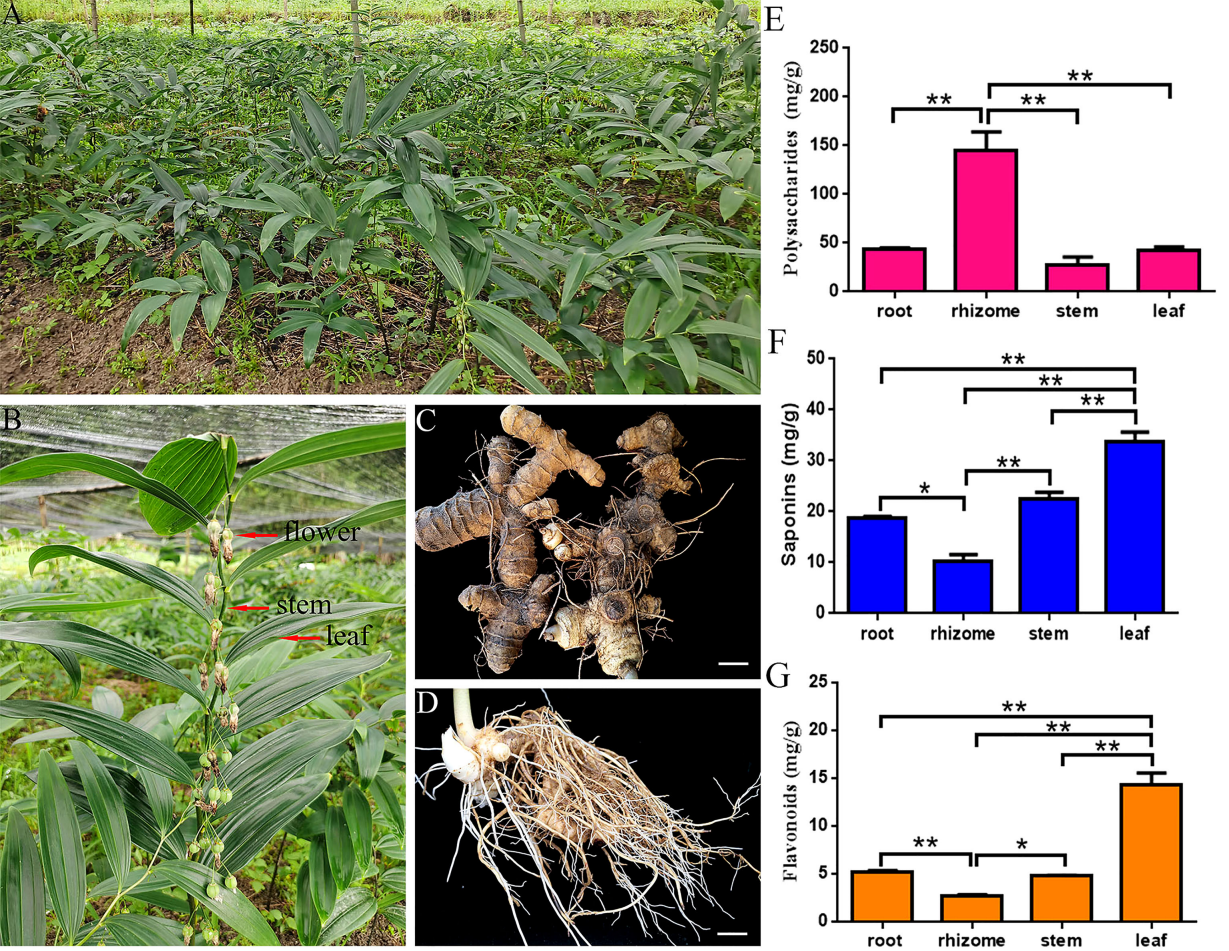
Representative photographs and physiological-biochemical indices of *P. cyrtonema.* **(A)** Mature *P. cyrtonema* in its natural habitat. **(B)** Flower, stems, and leaves. **(C)** Rhizome. **(D)** Root. **(E)** Total polysaccharides content. **(F)** Total saponins content. **(G)** Total flavonoids content. Bars = 1 cm. “**” and “*” represent significance at the 0.01 and 0.05 level, respectively.

*P. cyrtonema* was obtained from Wuyi Mountains, Nanping, Fujian, China (E117°16′12′′, N27°4′25′′). The root, rhizome, stem, and leaf were collected from the same three plants with three biological replicates (**Figures 1A–D**). Samples were cleaned with ultrapure water and separately packaged in Ziplock bags or tubes. The roots, rhizomes, stems, and leaves of each were frozen in liquid nitrogen and then quickly stored in a -80°C freezer for transcriptome sequencing and metabolic identification.

### Widely-targeted metabolomic analysis

The roots, rhizomes, stems, and leaves with three biological replicates were collected and then freeze-dried in a vacuum freeze-dryer (Scientz-100F). The sample powder was produced by a grinding mill (MM 400, Retsch) for 1.5 min at 30 Hz. One hundred milligrams of powder was dissolved in 1.2 mL of 70% methanol, vortexed 6 times, and placed overnight in a 4°C refrigerator. The supernatant obtained by centrifugation (12,000 rpm for 10 min) was filtered through a 0.22-µm membrane before ultraperformance liquid chromatography-tandem mass spectrometry (UPLC-MS/MS) analysis (Fraga et al., 2010). The analytical conditions of UPLC were as follows: column: Agilent SB-C18 (1.8 µm, 2.1 mm*100 mm); mobile phase: water (0.1% formic acid): acetonitrile (0.1% formic acid); gradient program: 95:5 (v/v) at 0 min, 5:95 (v/v) at 9 min, 5:95 (v/v) at 10 min, 95:5 (v/v) at 11.10 min, 95:5 (v/v) at 14 min; flow rate: 0.35 mL/min; column temperature: 40°C; injection volume: 4 μl. The detection parameters were set as follows: ion source; turbo spray; collision gas: GSI (50 psi)/GSII (60 psi)/curtain gas (25 psi); ion spray voltage: 5.5 kV (electrospray ionization, ESI^+^)/4.5 kV (ESI^-^), and ion source temperature, 550°C (Xia et al., 2009). Triple quadrupole scans were acquired as multiple reaction monitoring (MRM) experiments with optimized declustering potential and collision energy for each individual MRM transitions. The mass range was set between 50 and 1000 m/z, and product ions of each metabolite ion were scanned from 50 to 1,000 Da. After obtaining the metabolite spectrum analysis data, the peak area integration was performed on the mass spectrum peaks of all substances, and the integral correction was performed on the mass spectrum peaks of the same metabolite in different samples. Mass spectrum data was conducted with Analyst software (version 1.6.1) (Xia et al., 2009). Principal component analysis (PCA) and hierarchical cluster analysis (HCA) were performed and plotted by the R package (Luu et al., 2017). Differentially accumulated metabolites (DAMs) were identified with thresholds of log_2_|(fold change)|≥2, *p* value < 0.05, and variable importance in project (VIP) ≥ 1 and then annotated and enriched by the Kyoto Encyclopedia of Genes and Genomes (KEGG) compound and pathway databases.

### Alpha-glucosidase inhibitory activity experiment

To detect the α-glucosidase inhibition activity, a mixture of 50 μL of α-glucosidase solution (1.25 U/mL) and 50 μL of inhibitor (acarbose, 4-hydroxymandelonitrile, or eleutheroside E) was first incubated at 37°C for 10 min, and then second incubated at 37°C for 20 min after the addition of 150 μL of PNPG solution (5 mmol/L). The reaction was stopped by heating for 10 min at 100°C after the addition of 1 mL of Na_2_CO_3_ (1 mol/L). The reaction solution was cooled to room temperature and then measured at 405 nm using a microplate reader (OPTIMA S/N413-3915). Acarbose and phosphate buffer (pH 6.8, 0.1 mol/L) solutions were used as the positive and blank controls, respectively. A control reaction was used, in which the α-glucosidase solution was replaced with phosphate buffer solution. A negative reaction was used, in which the inhibitor was replaced with the phosphate buffer solution. Inhibitory concentrations of 50% (IC_50_) were analyzed by GraphPad Prism (version 9.0.0.121) using the specific calculation formula (Y=Bottom+ (Top-Bottom)/(1 + 10^((LogIC_50_-X)*HillSlope))). The inhibition percentage of α-glucosidase was calculated by the following formula:

α-glucosidase inhibition rate (%) = [1-(*OD_A_*-*OD_a_*)/(*OD_B_*-*OD_b_*)]×100%,

where *OD_A_* = activity with inhibitor (acarbose, 4-hydroxymandelonitrile, or eleutheroside E), *OD_a_* = activity with control reaction (without α-glucosidase solution), *OD_B_* = activity with negative control (without inhibitor), and *OD_b_* = activity with blank control (with phosphate buffer). One unit of enzyme inhibition was expressed by the weight of the IC_50_ value per milliliter.

### Library construction and sequencing

Total RNA was extracted using TRIzol^®^ Reagent (Invitrogen, Waltham, MA, USA) and then subjected to an Agilent 2100 Bioanalyzer (Agilent, Santa Clara, CA, USA) and electrophoresis for qualitative and quantitative analysis. The cDNA library was constructed by using the cDNA Library Construction Kit (Takara Biomedical Technology Co., Ltd., San Jose, CA, USA) and then sequenced on the Illumina platform (Illumina, San Diego, CA, USA) for mRNA sequencing. Total RNA or purified small RNA was ligated, reverse transcribed, amplified, and purified by TruSeq^®^ Small RNA sample preparation guide (Illumina, San Diego, CA) to generate a cDNA library and then sequenced on the Illumina platform for miRNA sequencing.

### *de novo* assembly, annotation and differential expression analysis

Default parameters were used to remove the low-quality sequences and adapter sequences by using FastQC software (version 11.0.4) (Brown et al., 2017). Trinity software (version 2.1.1) was used to generate a complete reference sequence (Grabherr et al., 2013). Assembled transcripts were assessed using the Benchmarking Universal Single Copy Orthologs (BUSCO) databases with viridiplantae_odb10 (Simão et al., 2015). The fragments per kilobase million (FPKM) value of the expression levels of transcripts was calculated using RNA-Seq by Expectation Maximization (RSEM) software (version 1.2.26) (Li and Dewey, 2011). The unique transcripts were annotated to the nonredundant (NR) database using basic local alignment search toolX (BlastX) software (version 2.10) with a threshold of E-value ≤10−5 (Camacho et al., 2009). Classification of Gene Ontology (GO) and KEGG was carried out using Blast2GO software (version 2.5) and KEGG automatic annotation sever, respectively (Conesa et al., 2005). Transcription factors (TFs) were predicted by PlantRegMap incorporated into the PlantTFDB (version 4.0) server with an E-value of 1e-5 (Jin et al., 2017). Differential expression analysis was identified by the DESeq R package (version 1.10.1) with thresholds of fold change>2 and *p* value<0.05 (Love et al., 2014). GO and KEGG enrichment analyses of DEGs were carried out by the topGO R package (version 3.8) and the KEGG orthology based annotation system (KOBAS) (version 3.0) (Zhou and Su, 2007; Xie et al., 2011).

### miRNA identification and target gene prediction

We filtered repetitive sequences and other ncRNAs (rRNA, tRNA, snRNA, and snoRNA) by comparison with the Silva, GtRNAdb, Rfam, and Repbase databases (Chan and Lowe, 2009). The remaining sequences were used to identify known miRNAs and novel miRNAs by comparison with the miRBase database (Kozomara and Griffiths-Jones, 2014). The novel miRNA secondary structure was predicted and calculated by using RNAfold (version 2.1.7) and RNAfold tools (version 2.0), respectively (Denman, 1993). DEMs were identified by using the DESeq R package (version 1.10.1) with thresholds of *p* value<0.05 and log_2_|(fold change)|>1, and DEM target genes were predicted by using TargetFinder (version 1.6) based on sequences of known miRNAs and novel miRNAs (Kiełbasa et al., 2010; Love et al., 2014). The annotation databases for DEM target genes were as follows: GO, NR, Pfam, COG, Swiss-Prot, KEGG, and Eukaryotic orthologous groups (KOG) databases, and enriched into GO terms and KEGG pathways by topGO R packages (version 3.8) and KOBAS (version 3.0) (Zhou and Su, 2007; Xie et al., 2011).

### mRNA-miRNA interaction network analysis

mRNA-miRNA interaction network analysis was carried out based on the results of DEGs and DEMs. Each DEG regulated by DEMs was obtained using TargetScan software (version 4.0) (Friedman et al., 2009). The targeting relationship between miRNA and mRNA was identified by a two-tailed Fisher’s exact test with functional divergence ratio (FDR)<0.05 and log_2_|(fold change)|>1 and then visualized using Cytoscape (version 3.7.2) (Doncheva et al., 2019). Selected DEGs were further subjected to functional analysis by GO and KEGG enrichment (Zhou and Su, 2007; Xie et al., 2011).

### Real-time quantitative polymerase chain reaction (qRT-PCR) validation

Total RNA was extracted with TRIzol (Takara, Tokyo, Japan) and purified with an EasyPure^®^ RNA Purification Kit (TransGen Biotech, Beijing, China). qRT-PCR was performed in three biological replicates using TransScript^®^ Green One-Step qRT-PCR SuperMix (TransGen Biotech, China). All miRNAs were normalized to U6 as an internal loading control, and glyceraldehyde-3-phosphate dehydrogenase (GAPDH) was used as an internal reference gene for mRNAs (**Table S1**).

## Results

### Phenotypic analysis of *P. cyrtonema* in different tissues

To preliminarily explore the content of effective active ingredients, we detected the content of polysaccharides, flavonoids, and saponins in different *P. cyrtonema* tissues by ultraviolet-visible (UV-Vis) spectrophotometry. The highest polysaccharides content reached 145.52 mg/g in rhizome, which was 3.34-, 5.28-, and 3.45-fold higher than the polysaccharides content of the root (43.51 mg/g), stem (27.58 mg/g), and leaf (42.23 mg/g), respectively (**Figure 1E**). In addition, the content of saponins and flavonoids in leaves (18.87 mg/g, 14.35 mg/g) was higher than the content of saponins and flavonoids in roots (9.39 mg/g, 5.18 mg/g), rhizomes (9.09 mg/g, 2.65 mg/g), and stems (12.55 mg/g, 4.75 mg/g), respectively (**Figures 1F, G**). These results showed that polysaccharides were the main active ingredients in *P. cyrtonema* as a result of the extremely high content.

### Metabolic profiles of *P. cyrtonema* in different tissues

**Figure 2:**
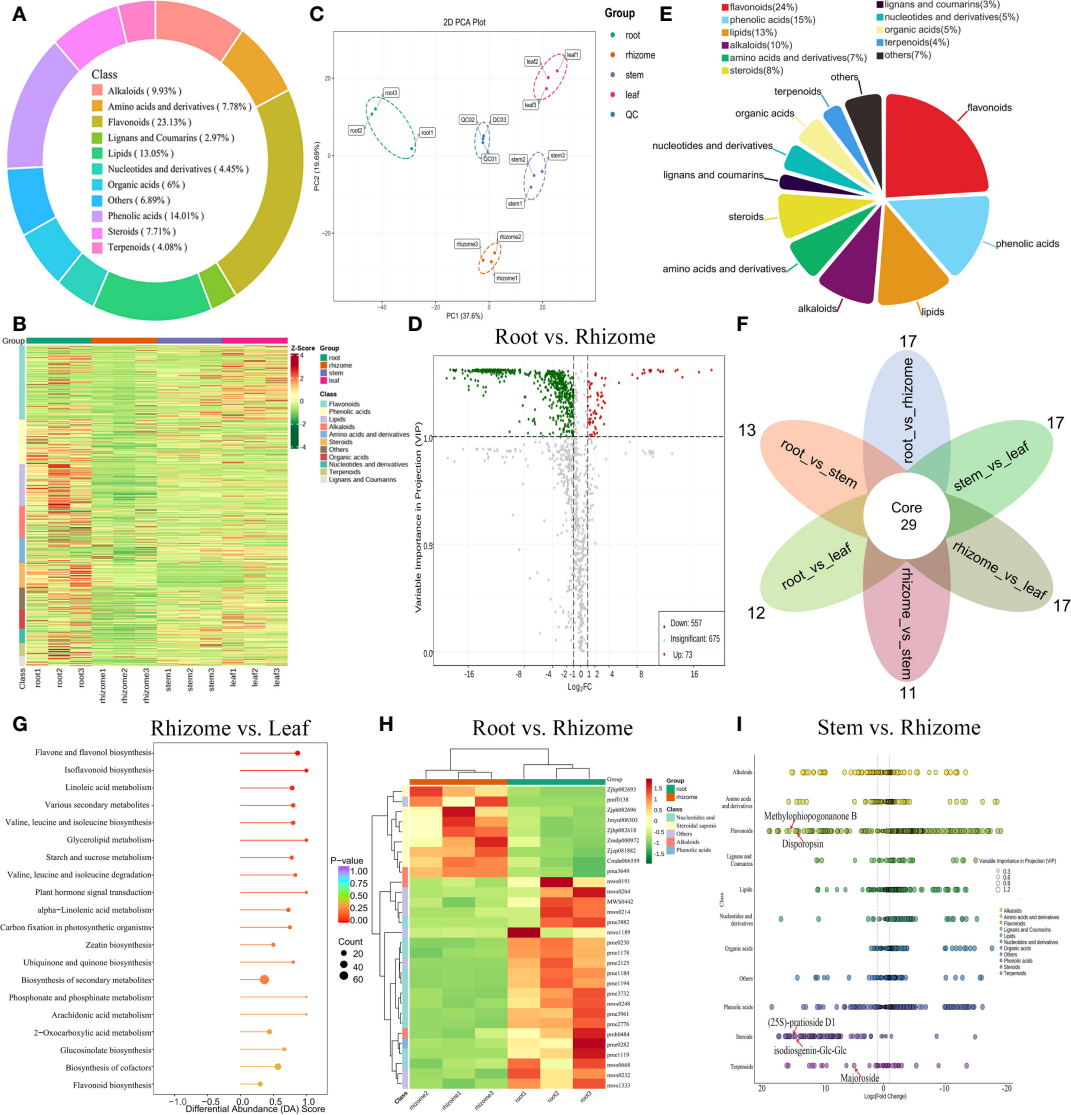
Metabolic profiles of *P. cyrtonema* in different tissues. **(A)** Ring diagram of metabolite component categories. **(B)** HCA of metabolites. **(C)** PCA of roots, rhizomes, stems, and leaves. **(D)** Volcano diagrams showing the DAMs in the root vs rhizome group. **(E)** Pie chart showing the percentage of each class of DAMs. **(F)** Venn diagram of DAMs shared among all groups. **(G)** Enrichment analysis of DAMs in the rhizome vs leaf group. **(H)** Top 30-fold changes in DAMs in the root vs rhizome group. **(I)** Scatter plot of DAMs in the stem vs rhizome group.

A total of 1,349 metabolites were successfully detected in the roots, rhizomes, stems, and leaves of *P. cyrtonema* by UPLC-MS/MS, including 312 flavonoids (23.13%), 189 phenolic acids (14.01%), 176 lipids (13.05%), 134 alkaloids (9.93%), 105 amino acids and derivatives (7.78%), 104 steroids (7.71%), and 40 lignans and coumarins (2.97%) (**Figure 2A**). PCA and HCA revealed that the metabolic profiles from *P. cyrtonema* in root, rhizome, stem, and leaf tissues exhibited different levels (**Figures 2B, C**). By pairwise comparison, we identified a total of 630 differentially accumulated metabolites (DAMs) (73 up- and 557 downregulated) between the root and rhizome, 769 DAMs (247 up- and 522 downregulated) between the root and stem, 786 DAMs (308 up- and 478 downregulated) between the root and leaf, 652 DAMs (438 up- and 214 downregulated) between the rhizome and stem, 703 DAMs (527 up- and 176 downregulated) between the rhizome and leaf, and 480 DAMs (318 up- and 162 downregulated) between the stem and leaf (**Figure 2D**; **Figure S1**; **Table S2**). Additionally, these DAMs were classified into eleven groups, with the largest number of metabolites falling under flavonoids, followed by phenolic acids, lipid, and alkaloids (**Figure 2E**). Venn diagram analysis revealed that 29 DAMs were shared among all groups, whereas 17, 13, 12, 11, 17, and 17 DAMs were unique to root_vs_rhizome, root_vs_stem, root_vs_leaf, rhizome_vs_stem, rhizome_vs_leaf, and stem_vs_leaf, respectively (**Figure 2F**). These DAMs were mainly enriched in “phenylalanine metabolism”, “biosynthesis of secondary metabolites”, “2-oxocarboxylic acid metabolism”, and “flavone and flavonol biosynthesis” (**Figure 2G**; **Figure S2**). Most secondary metabolites in nucleotides and derivatives, alkaloid, and phenolic acid classes were also upregulated, whereas most metabolites in the steroidal saponins class were downregulated in roots compared to rhizomes (**Figure 2H**). DAMs related to flavonoids, phenolic acids, and amino acids and derivatives were found to accumulate significantly in roots vs stems, roots vs leaves, and stems vs leaves (**Figure S2**). In the comparison of stem vs rhizome, most flavonoids, terpenoids, and steroidal saponins were significantly upregulated, including isoophiopogonanone B (17.20-fold increase), methylophiopogonanone B (17.10-fold increase), disogenin-Glc-Glc-Glc-Glc-Xyl (16.87-fold increase), disporopsin (15.68-fold increase), (25S)-pratioside D1 (15.93-fold increase), isodiosgenin-Glc-Glc (15.23-fold increase), majoroside (4.75-fold increase), and β-eudesmol (1.52-fold increase) (**Figure 2I**). Notably, we also found that steroids, flavonoids, phenolic acids, alkaloids, lignans and coumarins, and amino acids and derivatives were significantly upregulated in the comparison of leaf vs rhizome, including eleutheroside E (56.81-fold increase), gentrogenin-Glc (20.89-fold increase), pennogenin-Glc (16.35-fold increase), (25S)-pratioside D1 (15.68-fold increase), disporopsin (15.68-fold increase), luteolin-7-O-(6’’-sinapoyl) glucoside (14.09-fold increase), raspberryketone glucoside (1.29-fold increase), β-eudesmol (1.47-fold increase), and β-sitosterol (1.13-fold increase) (**Figure S3A**). Additionally, most saccharides except D-mannose were significantly updowned, including D-glucono-1,5-lactone, arabinose, cellobiose, maltotetraose, panose, threose, trehalose, and glucurono-6,3-lactone in the rhizome compared with root (**Figure S3B**). These results indicated that flavonoids and steroidal saponins, in addition to polysaccharides, are important active ingredients affecting the medicinal value of *P. cyrtonema*.

### Alpha-glucosidase inhibitory activity

**Figure 3:**
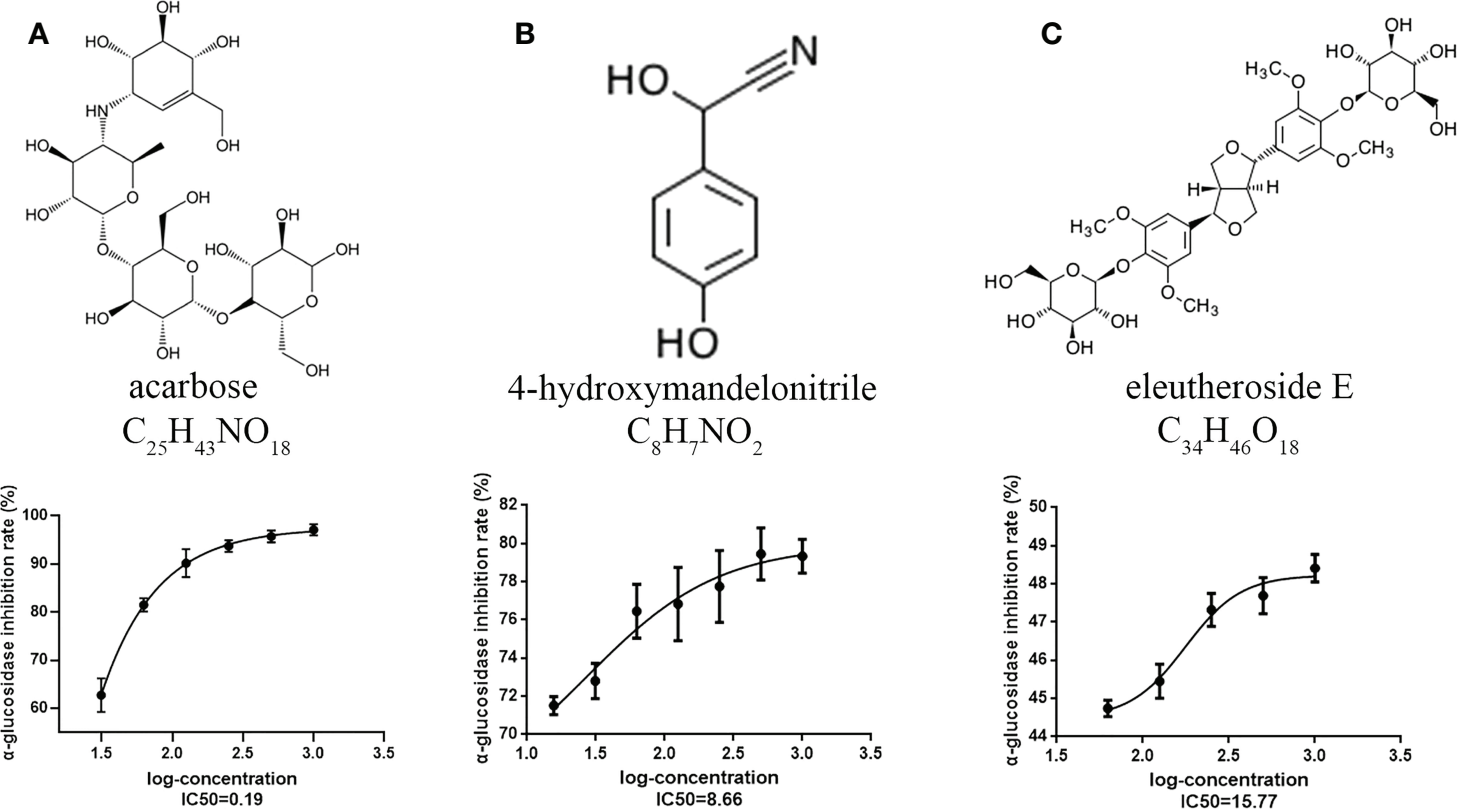
Alpha-glucosidase inhibitory activity assay of DAMs. **(A)** Acarbose. **(B)** 4-hydroxymandelonitrile. **(C)** Eleutheroside E. Each value represents the mean ± SD, and error bars represent significant differences (n = 3, *P < 0.05*).

To confirm the results of the metabolome analysis, an α-glucosidase inhibitory activity assay was conducted on raspberryketone glucoside, β-sitosterol, majoroside, 4-hydroxymandelonitrile, β-eudesmol, and eleutheroside E selected based on the metabolic changes and differential analysis. The α-glucosidase inhibitory activity results showed that two of six acarbose, 4-hydroxymandelonitrile, and eleutheroside E reduced the stability of the α-D-glucoside bond with IC_50_ values of 8.66 μM, 15.77 μM, and 0.19 μM, respectively. We also found that the highest inhibition of 4-hydroxymandelonitrile and eleutheroside E was 79.0% and 47.41% at a concentration of 1.0 mM, which was lower than that of acarbose at 1.0 mM (98.45%) (**Figure 3**). The results showed that 4-hydroxymandelonitrile have a relatively good inhibitory effect on α-glucosidase, indicating that it may play a role in hypoglycemic functions.

### Analysis of transcriptomic profiles and differentially expressed genes

**Figure 4:**
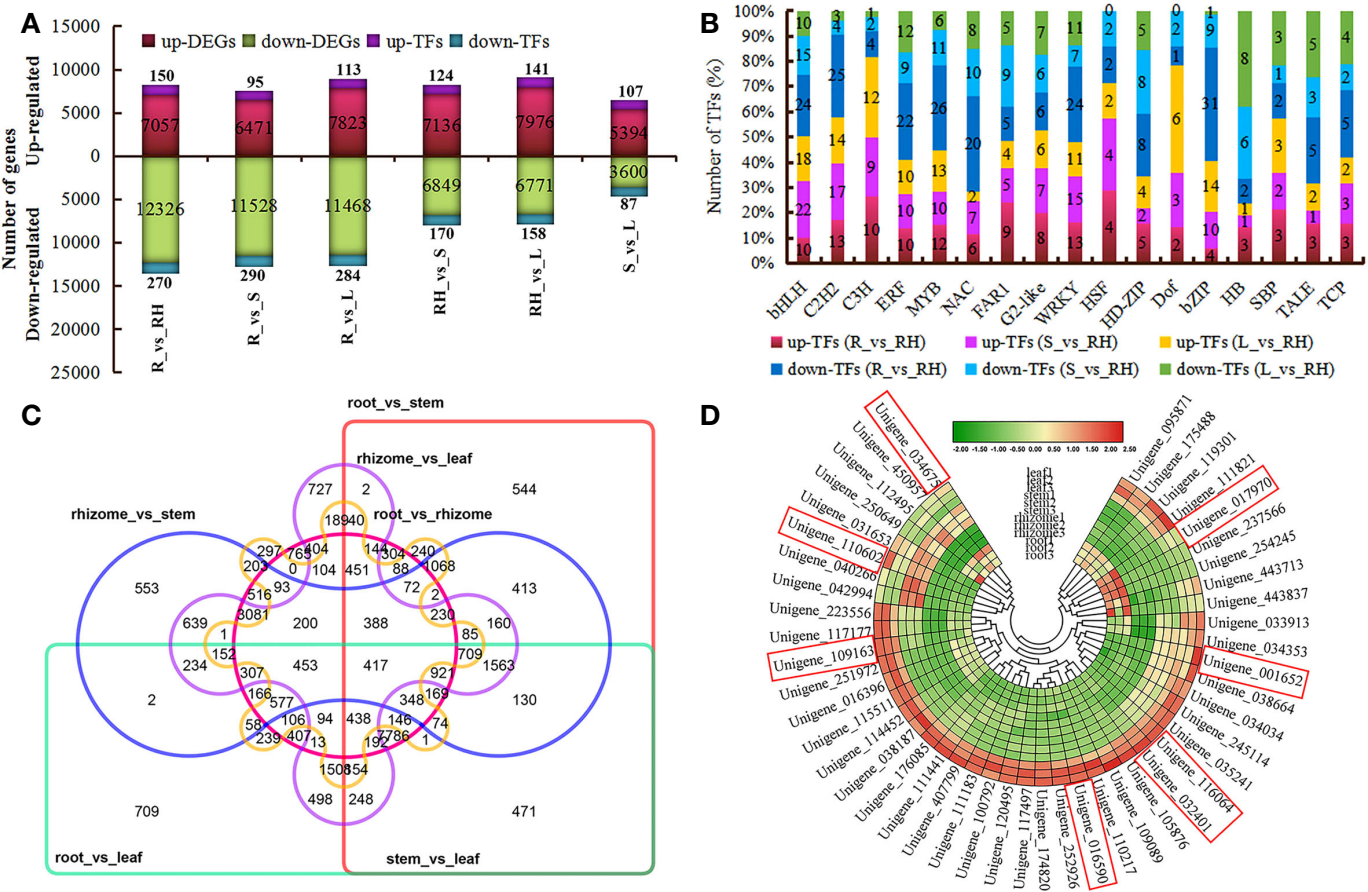
Differential gene expression by pairwise comparison. **(A)** Statistics of up- and downregulated unigenes or TFs. **(B)** The number of genes from different TF families showing up- or downregulation at different tissues. **(C)** Venn diagram of DEGs shared among all groups. **(D)** Heatmap showing DEGs involved in terpenoids and flavonoids in the leaf compared with the rhizome.

A total of 92.95 Gb clean data were obtained with an average of 25,819,386 clean reads and 7,745,815,700 clean bases per sample. A total of 53,336 unigenes were generated after *de novo* assembly using Trinity software (version 2.5.1) with a unigene N50 of 2,118 bp. Approximately 94.6% of the conserved genes were complete in the *de novo* assembled transcripts by BUSCO analysis (**Table S3**). Out of 43,189 (80.98%) unigenes were annotated to TrEMBL (42,042, 78.82%), Swiss-Prot (29,597, 55.49%), Nr (42,831, 80.30%), eggNOG (36,097, 67.68%), GO (3,5556, 66.66%), KOG (24,507, 45.95%), COG (14,003, 26.25%), KEGG (29,749, 55.78%), and Pfam (33,801, 63.37%) (**Table S4**). Pairwise comparison analysis revealed a total of 19,383 (including 420 TF-encoding genes), 17,999 (including 385 TF-encoding genes), 19,291 (including 397 TF-encoding genes), 13,985 (including 294 TF-encoding genes), 14,747 (including 299 TF-encoding genes), and 8,994 (including 194 TF-encoding genes) DEGs in root vs rhizome, root vs stem, root vs leaf, rhizome vs stem, rhizome vs leaf, and stem vs leaf, respectively (**Figure 4A**; **Table S5**). Several TFs associated with flavonoids, sesquiterpenoid, and triterpenoid biosynthesis, such as Dof, C3H, MYB, HSF, WRKY, and bHLH, were expressed at significantly higher levels in rhizomes than in other tissues (**Figure 4B**). Some members of the WRKY family showed high gene expression levels in leaves, such as *PcWRKY13* (Unigene_154754), *PcWRKY4* (Unigene_227001), *PcWRKY55* (Unigene_111966), *PcWRKY33* (Unigene_206697), *PcWRKY71* (Unigene_037336), and *PcWRKY51* (Unigene_087798), which may regulate starch and sucrose metabolism. Further analysis revealed that 417 DEGs were shared by all groups, whereas 765, 544, 709, 553, 727, and 297 DEGs were unique to root vs rhizome, root vs stem, root vs leaf, rhizome vs stem, rhizome vs leaf, and stem vs leaf, respectively (**Figure 4C**). KEGG pathway enrichment analysis showed that rhizome-specific DEGs were significantly upregulated in polysaccharides biosynthesis compared with other tissues, including *PcGN1* (Unigene_169058), *PcFK* (Unigene_169962), *PcsacA* (Unigene_096419), *PcSUS* (Unigene_168538), *PcAMY* (Unigene_173855), *PcSPP* (Unigene_203438), and *PcPYG* (Unigene_219490) (**Figure S4**). Forty-seven DEGs involved in terpenoid and flavonoids pathways in the leaf group exhibited higher expression levels and were upregulated compared with the rhizome, including *PcPGT1* (Unigene_016590), *PcANR* (Unigene_017970), *PcHCT* (Unigene_032401), *PcF3H* (Unigene_109163), *PcFLS* (Unigene_110602), *PcNES1* (Unigene_001652), *PcCYP71D55* (Unigene_034675), and *PcSQLE* (Unigene_116064) (**Figure 4D**).

### Hub gene identification related to the accumulation of the main bioactive ingredients

**Figure 5:**
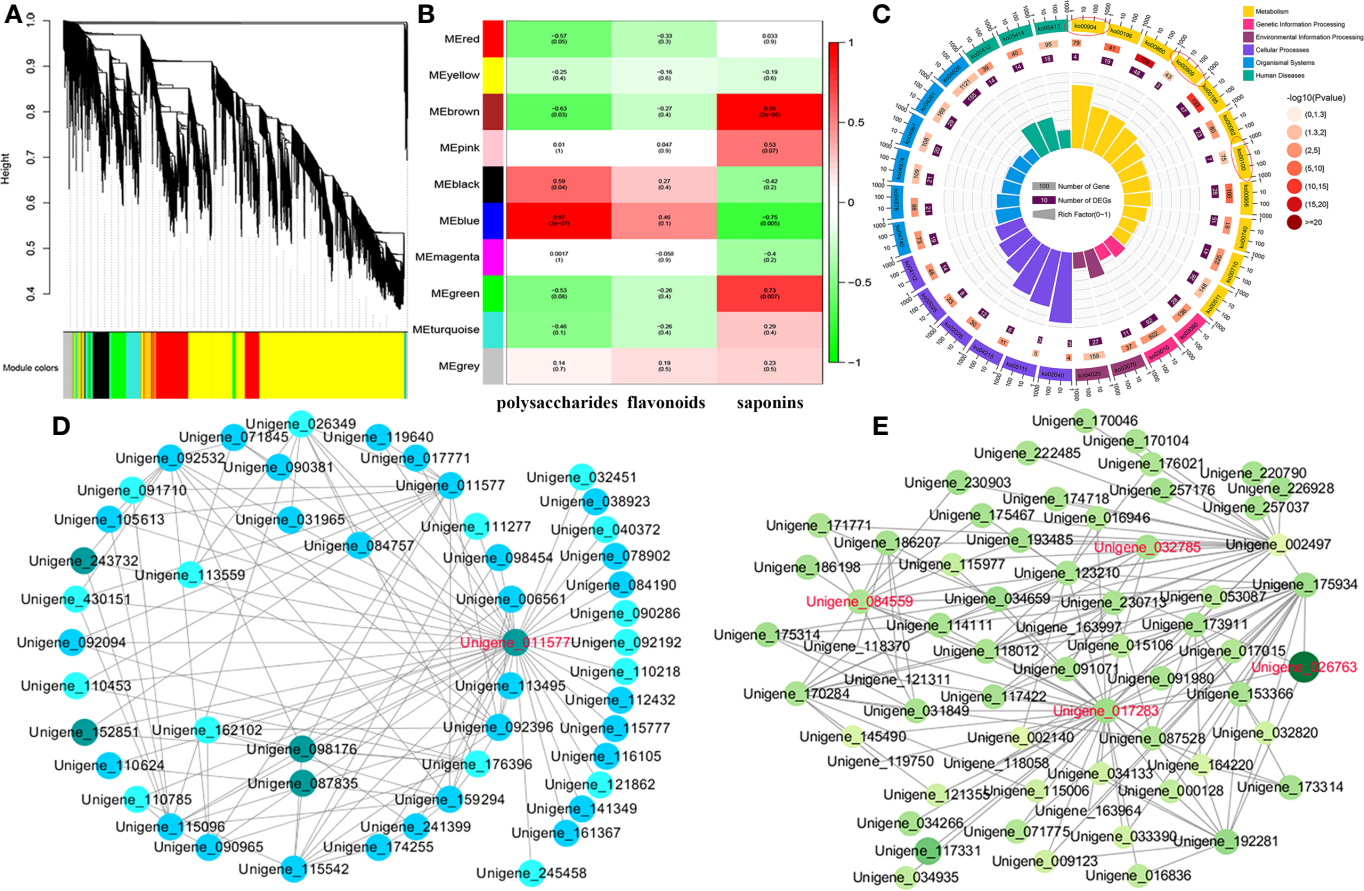
Hub genes related to polysaccharides, saponins, and flavonoids accumulation. **(A)** Cluster dendrogram of DEGs for WGCNA analysis. **(B)** Gene modules associated with the content of polysaccharides, flavonoids, and saponins. **(C)** Enrichment analysis of DEGs in the brown and green modules. **(D)** Co-expression network revealing the hub genes related to saponins content in the brown and green modules. **(E)** Co-expression network revealing the hub genes related to polysaccharides content in the blue module.

To identify the hub genes associated with total polysaccharides, flavonoids, and saponins, we performed WGCNA for the RNA-seq data of 12 samples, and 10 coexpression modules with red (2,437 genes), blue (5,729 genes), turquoise (6,426 genes), black (2,293 genes), brown (5,525 genes), pink (870 genes), yellow (3,838 genes), magenta (816 genes), green (3,302 genes), and gray (1,748 uncorrelated genes) were generated (**Figure 5A**). Notably, we found that two modules, brown (r = 0.95 and *p* value = 2E-6) and green (r = 0.73 and *p* value = 0.007), were significantly correlated with saponins content (**Figure 5B**). KEGG analysis revealed that the following pathways were highly enriched in the above two modules: steroid (ko00100), diterpenoid (ko00904), sesquiterpenoid and triterpenoid (ko00909) biosynthesis, including *PcSQLE* (Unigene_116064), *PcGGPS* (Unigene_030422), *PcCYP71D55* (Unigene_117762), *PcLIPA* (Unigene_030602), *PcSMO2* (Unigene_176527), *PcIPK* (Unigene_017694), *PcSMT1* (Unigene_246330), and *PcEBP* (Unigene_037516) (**Figure 5C**; **Figures S5**-**S7**). We captured one hub gene (*PcCYP71A1*, Unigene_011577) that shared 41 edges with other candidate genes. Studies have shown that CYP71, CYP77, CYP86, CYP89, CYP90, and CYP91 family members participate in the structural modification of triterpenoid saponins. We speculated that *PcCYP71A1* may be a hub gene related to saponins accumulation (**Figure 5D**). Furthermore, the blue module was identified as having a significantly high association with total polysaccharides content (r = 0.97 and *p* value = 3E-7), and most genes from this module were enriched in amino sugar and nucleotide sugar metabolism, fructose and mannose metabolism, starch and sucrose metabolism, and glycolysis, including *PcFK* (Unigene_000991), *PcHK* (Unigene_039450), *PcPFK* (Unigene_026763), *PcAMY* (Unigene_017283), *PcGN1* (Unigene_032610), *PcsacA* (Unigene_084559), and *Pcβ-AMY4* (Unigene_032785) (**Figure 5E**). In addition, a total of 124 TFs were identified in the blue module, and we found that five MYB gene family members may be correlated with flavonoids content, including *PcMYB3* (Unigene_256430), *PcMYB97* (Unigene_032505), *PcMYB102* (Unigene_182574), *PcMYB33* (Unigene_226806), and *PcMYB61* (Unigene_173029) (**Table S6**).

### Identification and expression analysis of miRNAs and their targets

**Figure 6:**
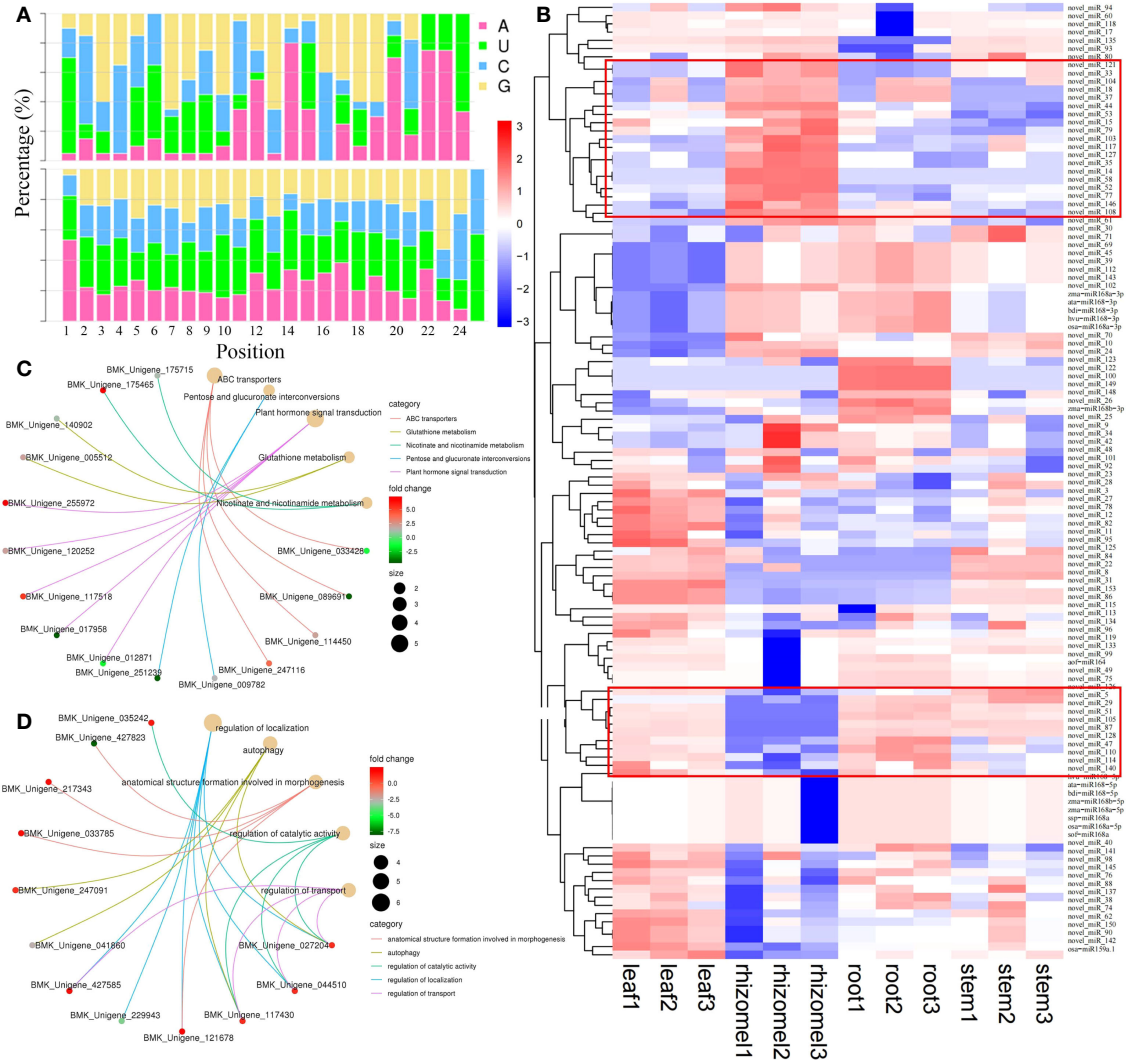
Expression analysis of miRNAs and their targets. **(A)** Base distribution sites of known and novel miRNAs. **(B)** Heatmap of miRNAs visualizing the changes in the expression profiles in different tissues. **(C)** Enrichment analysis of miRNA target genes in the root vs. rhizome group. **(D)** GO enrichment analysis of miRNA target genes in the root vs. leaf group.

Approximately 180.12 Mb of small RNA clean reads were mapped to a known miRNA database and represented a total of 169 miRNAs (20 known and 149 novel miRNAs) (**Figure 6A**; **Table S7**). miR172 was the largest family, with 15 members, followed by miR167_1 (10), miR166 (9), and miR395 (9) (**Table S8**). Notably, osa_miR159a showed high expression with an average FPKM of 297,083, followed by novel_miR24 (40,638), novel_miR24 (33,635), and novel_miR112 (30,768). By pairwise comparison, we identified a total of 50 (25 up and 25 down), 50 (25 up and 25 down), 62 (32 up and 30 down), 74 (40 up and 34 down), 57 (26 up and 31 down), and 79 (39 up and 40 down) DEMs in root vs rhizome, stem vs leaf, root vs stem, root vs leaf, rhizome vs stem, and rhizome vs leaf, respectively (**Table S9**). Heatmap analysis indicated that the expression levels of novel_miR103, novel_miR14, novel_miR117, novel_miR52, novel_miR77, and novel_miR58 in the rhizome were significantly higher than those expression levels in other tissues, while novel_miR5, novel_miR29, novel_miR105, novel_miR49, novel_miR87, and novel_miR51 showed the opposite trend, indicating that these DEMs may play important roles in secondary metabolite accumulation (**Figure 6B**). We also identified a total of 3,432 target genes for all miRNAs, and out of 878 target genes were functionally annotated to eight public databases (**Table S10**). Of these, 406, 383, 407, 348, 484, and 240 target genes were identified in root vs rhizome, root vs stem, root vs leaf, rhizome vs stem, rhizome vs leaf, and stem vs leaf, respectively (**Table S10**). KEGG analysis revealed that most of the genes were highly enriched in the following pathways: pentose and glucuronate interconversions, plant hormone signal transduction, nicotinate and nicotinamide metabolism, alpha-linolenic acid metabolism, and phenylpropanoid biosynthesis (**Figure 6C**; **Figure S8**). Most of the GO terms were enriched in the biological process category and were associated with developmental process, biological regulation, signaling, metabolic process, and cellular process (**Figure 6D**, **Figure S9**).

### Ming candidate miRNAs and mRNAs regulated metabolite biosynthesis by integrated analysis

**Figure 7:**
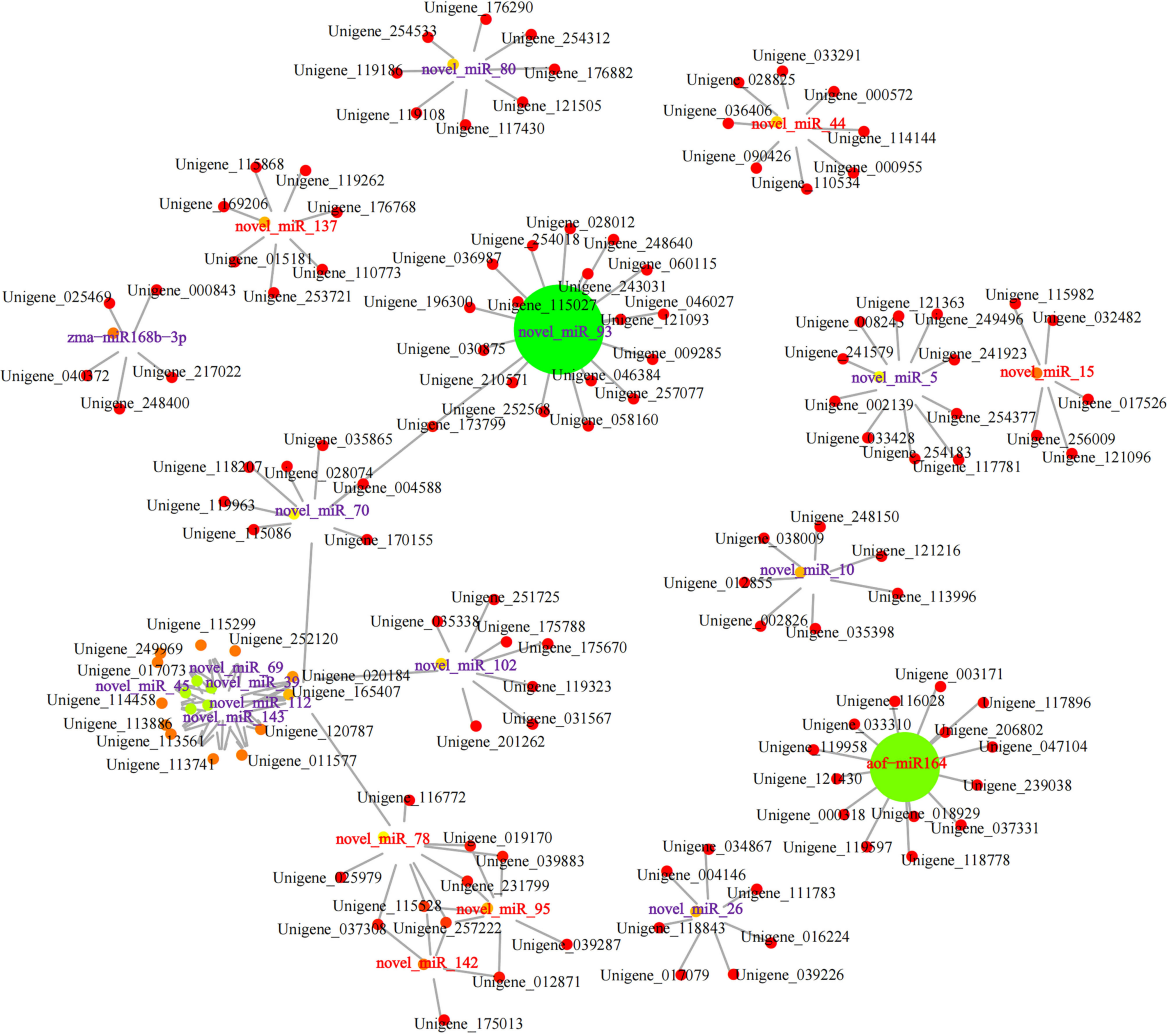
Network diagram of the targeting relationship between miRNAs and mRNAs. Red and purple fonts indicate the up- and downregulated miRNAs, respectively.

Integrative analyses showed that a total of 751 (24 upregulated miRNAs had 182 downregulated targets), 644 (27 upregulated miRNAs had 135 downregulated targets), 914(37 upregulated miRNAs had 162 downregulated targets), 562 (24 upregulated miRNAs had 115 downregulated targets), 855 (36 upregulated miRNAs had 178 downregulated targets), and 250 (23 upregulated miRNAs had 28 downregulated targets) miRNA-mRNA relationship pairs were identified in root vs rhizome, root vs stem, root vs leaf, rhizome vs stem, rhizome vs leaf, and stem vs leaf, respectively. A network of miRNA-mRNA interactions was constructed and visualized by using Cytoscape. For example, 127 mRNA targets were regulated by the top 20 miRNAs (upregulated: novel_miR142, novel_miR15, novel_miR78, novel_miR44, novel_miR137, novel_miR95, and aof_miR164; downregulated: novel_miR143, novel_miR80, novel_miR5, novel_miR93, novel_miR10, novel_miR102, novel_miR70, novel_miR26, novel_miR69, novel_miR45, novel_miR112, and novel_miR39) in the comparison of stem vs leaf (**Figure 7**). We found that these miRNA targets were related to terpenoid backbone biosynthesis, fatty acid degradation, alpha-linolenic acid metabolism, and plant hormone signal transduction, including *PcDXS* (Unigene_176882), *PcCYP71A1* (Unigene_011577), *PcADH5* (Unigene_252120), and *PcMYC2* (Unigene_114144). Furthermore, novel_miR148, novel_miR49, aof_miR164, and novel_miR75 were selected and negatively correlated with alpha-linolenic acid biosynthesis and MAPK signaling, including *PcFRK1* (Unigene_197690), *PcAOS* (Unigene_025136), *PcDELLA* (Unigene_231603), and *PcSPLA2* (Unigene_028081), in the subnetwork of root vs rhizome. These results indicated that these miRNAs may coordinately regulate the accumulation of the main bioactive ingredients and resistance in *P. cyrtonema*. qRT-PCR was carried out on ten DEGs or DEMs, including *PcFK*, novel_miR95, *PcSUS*, novel_miR143, *PcF3H*, aof_miR164, *PcAMY*, novel_miR14, *PcCYP71A1*, and novel_miR78. The qRT-PCR results were consistent with the expression levels of RNA-Seq, indicating that RNA-Seq analysis had high reliability (**Figure S10**).

## Discussion

*P. cyrtonema*, known as “longevity grasses”, is one of the traditional Chinese medicinal materials with homology of medicine and food published by the National Health Commission, as well as the traditional bulk medicinal materials in China (Zhao et al., 2018; Zhang et al., 2020; Zhang et al., 2021; Zhang et al., 2022). The dried rhizome of the herb *P. cyrtonema* has a variety of bioactive ingredients (polysaccharides, saponins, and flavonoids), which have been used mainly as vegetables, food, and medicine over the years with many beneficial effects, including treating cough, antiaging, and anti-inflammatory effects (Liao and Wu, 2021; Liu et al., 2021). Although a previous study showed that 637 metabolites were detected in *P. cyrtonema* seeds by LC/MS, no relevant in-depth study has reported the differences and expression profiles in DAMs in different tissues of *P. cyrtonema* (Liu et al., 2021). In this study, 349 metabolites were successfully detected in roots, rhizomes, stems, and leaves by UPLC-MS/MS. Of these, isoophiopogonanone B, disogenin-Glc-Glc-Glc-Glc-Xyl, (25S)-pratioside D1, 5,7-dihydroxy-6,8-dimethyl-3-(4’-hydroxy-3’-methoxybenzyl)chroman-4-one, disporopsin, isodiosgenin-Glc-Glc, and methylophiopogonanone B, which are associated with the intermediates in the flavonoids and saponins pathways, were significantly upregulated in the stem compared with the rhizome. Moreover, most saccharides, including D-dlucono-1,5-lactone, arabinose, cellobiose, maltotetraose, panose, threose, trehalose, and glucurono-6,3-lactone, which are involved upstream of the polysaccharides biosynthesis pathway, were found to be downregulated in the rhizome compared with the root. In addition, we also found that the expression levels of most genes related to polysaccharides accumulation were significantly upregulated in rhizome-specific DEGs, including *PcFK*, *PcsacA*, *PcGN1*, *PcAMY*, *PcPYG*, *PcSPP*, and *PcSUS*, suggesting that these metabolites may accumulate in large amounts upstream of the polysaccharides pathway by high expression of the above genes and serve as downstream substrates in the biosynthesis of various types of polysaccharides.

The transcriptome preliminarily clarified the metabolic pathways of active ingredients from *P. cyrtonema* and the expression of key enzyme genes by different tissues and seed germination stages; however, the biosynthetic mechanism is complex and is regulated by multiple dimensions, such as mRNA, miRNA and its targets (Wang et al., 2019; Fan et al., 2020; Liao et al., 2020; Liu et al., 2021). Although several genes involved in polysaccharides, saponins, and flavonoids biosynthesis were identified by conventional mRNA sequencing, the miRNA-mediated posttranscriptional regulatory mechanism is still unknown, which is obviously not enough to systematically elucidate the molecular mechanisms of active constituents. Of the 20 known and 149 novel miRNAs identified, osa_miR159a was found to be highly expressed in all tissues and was reported to mediate plant growth and resistance through phytohormone signaling pathways by targeting zinc finger transcription factors (Xu X et al., 2014). miR172i-targeted 4-coumarate-CoA ligase was also reported to regulate metabolic flux in the flavonoids pathway (Biswas et al., 2016). We speculated that miR172 is the largest family, with 15 members, and its family members with high expression in leaves may influence flavonoids accumulation. Novel_miR49, which is downregulated in the rhizome, has been identified as upregulated in the biosynthesis of phenolic compounds in tea plants (Jeyaraj et al., 2017). Three genes, *PcCYP71A1*, *PcMYC2*, and *PcSNRK2*, were identified to be negatively targeted by novel_miR112 in the comparison of stem vs leaf, which is associated with MAPK signaling and the terpenoid pathway. In addition, aof_miR164 was also found to target the *PcAOS*, *PcSPLA2*, *PcFRK1*, and *PcDELLA* genes and negatively regulate alpha-linolenic acid metabolism and the MAPK signaling pathway, resulting in a high content of the main active substances in *P. cyrtonema*.

Polysaccharides, saponins, and flavonoids are the main active ingredients of *P. cyrtonema* and have multiple pharmacological effects (Su et al., 2019). To better understand the regulatory mechanism of these active ingredients, WGCNA was performed to reveal the candidate genes involved in the accumulation of polysaccharides, saponins, and flavonoids. Several genes from brown and green modules were found to have a significant correlation with saponins content, including *PcSQLE*, which catalyzes squalene into oxidosqualene as a key enzyme of sterol biosynthesis (Kalra et al., 2013); *PcCYP71D55* and *PcCYP71A1*, which are related to the modification of triterpenoid saponins structure (Haralampidis et al., 2002); and *PcGGPS*, which participates in monoterpene production (Liu et al., 2022). These results suggested that upstream genes associated with monoterpenes, sesquiterpenes, and triterpenes are key enzyme genes for regulating the formation and modification of downstream steroids and saponins. Moreover, we captured four candidate genes in the blue module that were highly correlated with polysaccharides. For example, *PcPFK*, which catalyzes the first irreversible reaction in glycolysis (Ismail and Ul Hussain, 2017), *Pcβ-AMY4*, which is associated with starch-dependent maltose accumulation (Ma et al., 2022), *PcAMY*, which participates in starch and sucrose metabolism (Chen et al., 2021), and *PcsacA*, which is a key enzyme that is necessary for the metabolism of galactose (Aung et al., 2019). *ZmMYB42* regulates other genes related to the phenylpropanoid pathway, controlling the biosynthesis of flavonoids in *Arabidopsis thaliana* (Sonbol et al., 2009). *GbMYB2* negatively regulated flavonoids accumulation in *Ginkgo biloba* (Xu F et al., 2014). *VvMYB5b* upregulated a subset of anthocyanin structural genes, providing insight into flavonoids biosynthesis in a *Petunia* anthocyanin regulatory mutant (Deluc et al., 2008). Interestingly, *PcMYB3*, *PcMYB97*, *PcMYB102*, *PcMYB33*, and *PcMYB61* were correlated with flavonoids content in the blue module, suggesting that they may affect flavonoids biosynthesis in *P. cyrtonema* by regulating phenylpropanoid and anthocyanin pathways. These findings will facilitate in-depth research on the functions of the main active substances of these miRNAs and mRNAs for a comprehensive understanding of the roles of their pathological and biological regulation in *P. cyrtonema*.

## Conclusions

Phenotypic analysis showed that the polysaccharides content reached the maximum in the rhizome with 145.52 mg/g, which was significantly higher than the polysaccharides content of other tissues. Metabolomics data showed that alkaloids, flavonoids, steroids, and phenolic acids were the main active ingredients in *P. cyrtonema*, and most of these were significantly upregulated in rhizomes compared with other tissues, suggesting that they may contribute to the formation and accumulation of the main active ingredients in *P. cyrtonema*. To better understand the regulatory mechanism of these active ingredients, mRNA and WGCNA were combined to reveal several candidate genes involved in the accumulation of polysaccharides, saponins, and flavonoids, including *PcSQLE*, *PcCYP71D55*, *PcCYP71A1*, *PcGGPS*, *PcPFK*, *PcAMY*, *PcsacA*, *PcMYB3*, *PcMYB97*, *PcMYB102*, *PcMYB33*, and *PcMYB61*. Additionally, we found that aof_miR164, novel_miR14, novel_miR78, novel_miR95, and novel_miR143 may coordinately regulate the biosynthesis of secondary metabolites and resistance in *P. cyrtonema* by combining miRNA and mRNA analyses. These findings will facilitate in-depth research on the functions of the main active substances of these miRNAs and mRNAs for a comprehensive understanding of the roles of their pathological and biological regulation in *P. cyrtonema*.

## Data availability statement

The datasets presented in this study can be found in online repositories. The names of the repository/repositories and accession number(s) can be found below: https://ngdc.cncb.ac.cn, CRA007571.

## Author contributions

TX, CZ, and BL designed the study. MZ and JC processed and analyzed datasets. YC, TX, and BL prepared the manuscript. All authors contributed to the article and approved the submitted version.

## Funding

This work was supported by the Industry-University Cooperation Project of Fujian Science and Technology Department (No. 2016N5011; No. 2020N5008; No. 2021N5016) and Special Funding Project of Fujian Provincial Department of Finance (No. SC-299).

## Conflict of interest

The authors declare that the research was conducted in the absence of any commercial or financial relationships that could be construed as a potential conflict of interest.

## Publisher’s note

All claims expressed in this article are solely those of the authors and do not necessarily represent those of their affiliated organizations, or those of the publisher, the editors and the reviewers. Any product that may be evaluated in this article, or claim that may be made by its manufacturer, is not guaranteed or endorsed by the publisher.
