# Supplementary material for: Revealing the mechanisms of the bioactive ingredients accumulation in Polygonatum cyrtonema by multiomics analyses
Source: Front Plant Sci. 2022 Nov 16;13:1055721. doi: 10.3389/fpls.2022.1055721 (PMC9709641; doi:10.3389/fpls.2022.1055721)
Supplement: Supplementary file 1 [file DataSheet_1.doc]

**Supplementary Figures**

A B


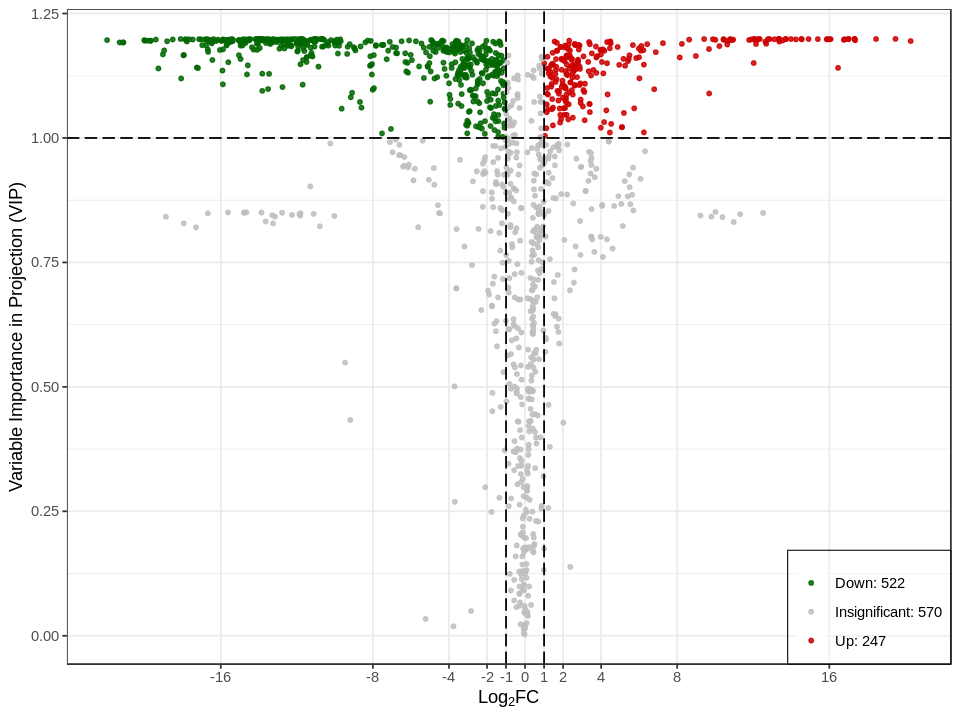

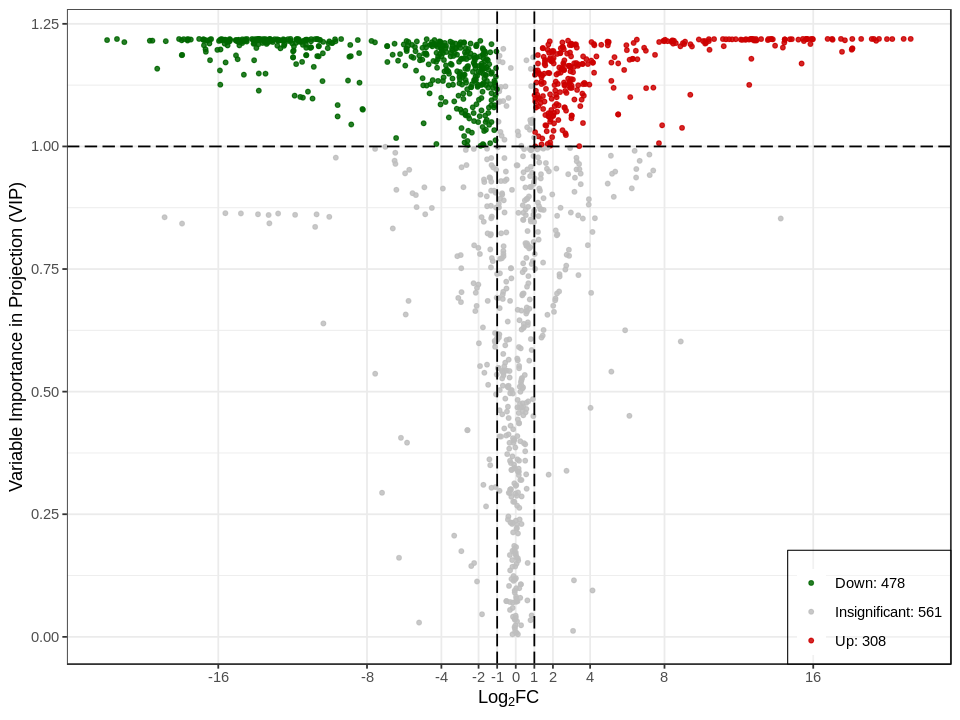


C D


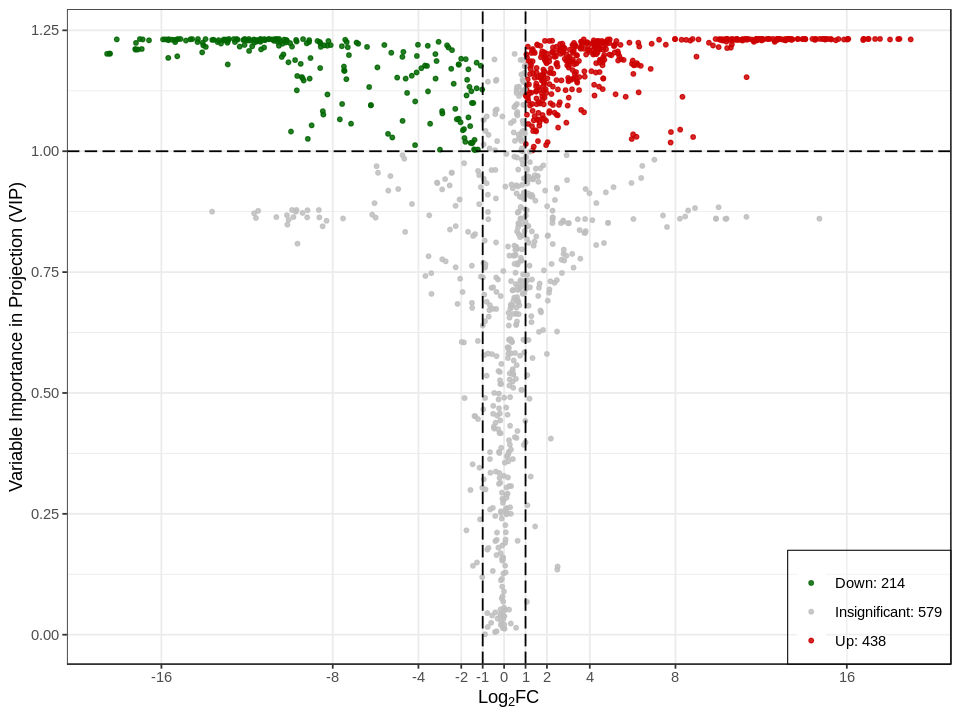

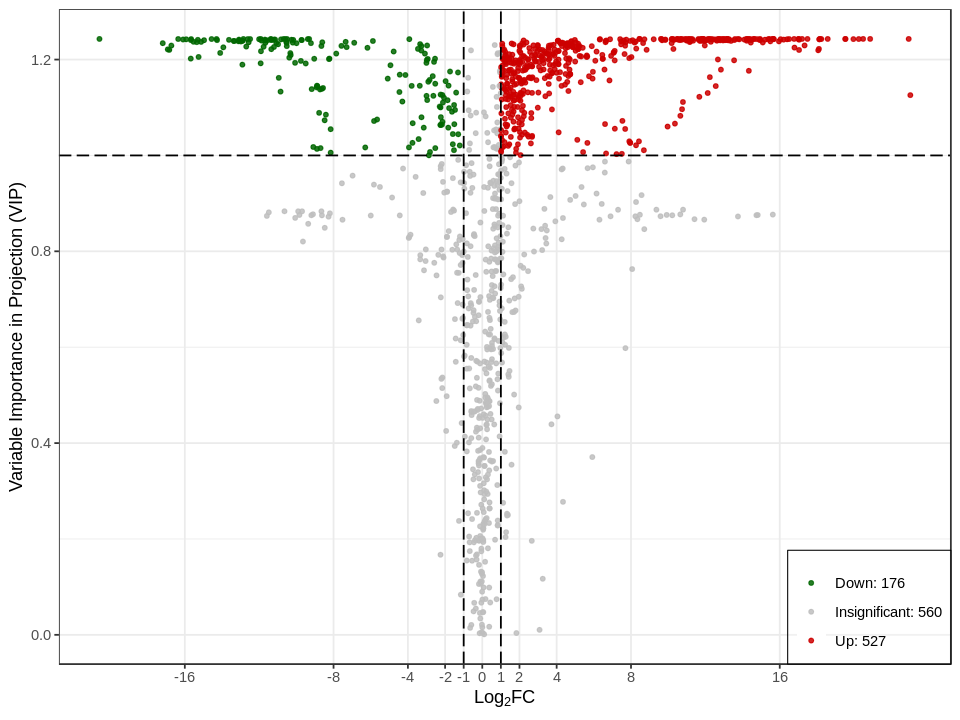


E


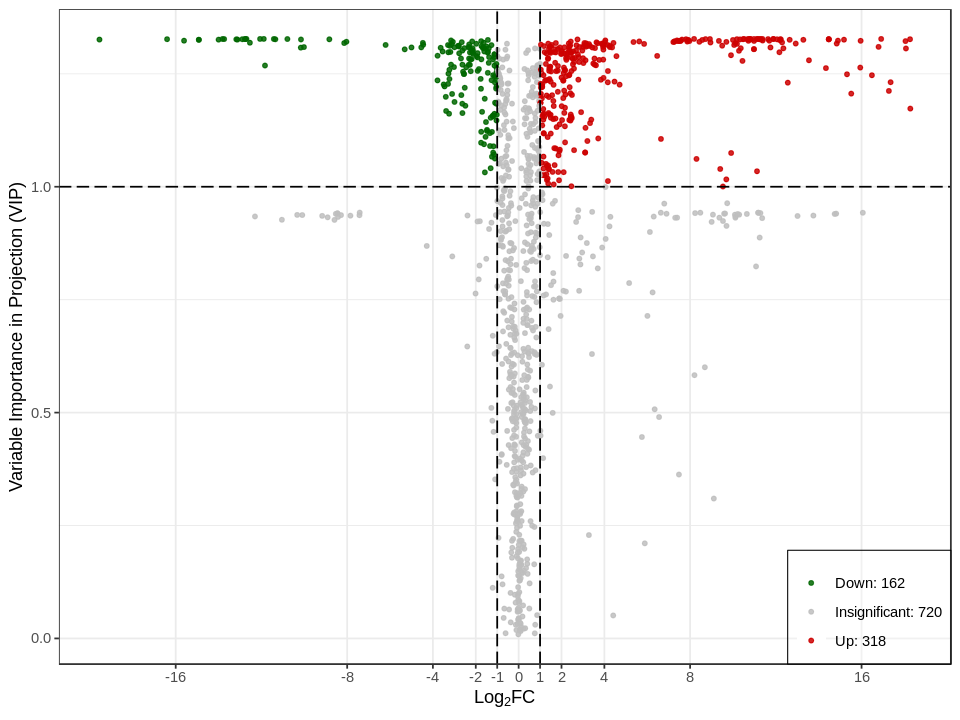


Figure S1 Volcano plot of differential metabolites for root vs. stem (A), root vs. leaf (B), rhizome vs. stem (C), rhizome vs. leaf (D), and stem vs. leaf (E), respectively. Each point in the figure represents a metabolite. Green points represent down-regulated metabolites, red points represent up-regulated metabolites, and gray points represent metabolites that were detected but not significantly different.

A B


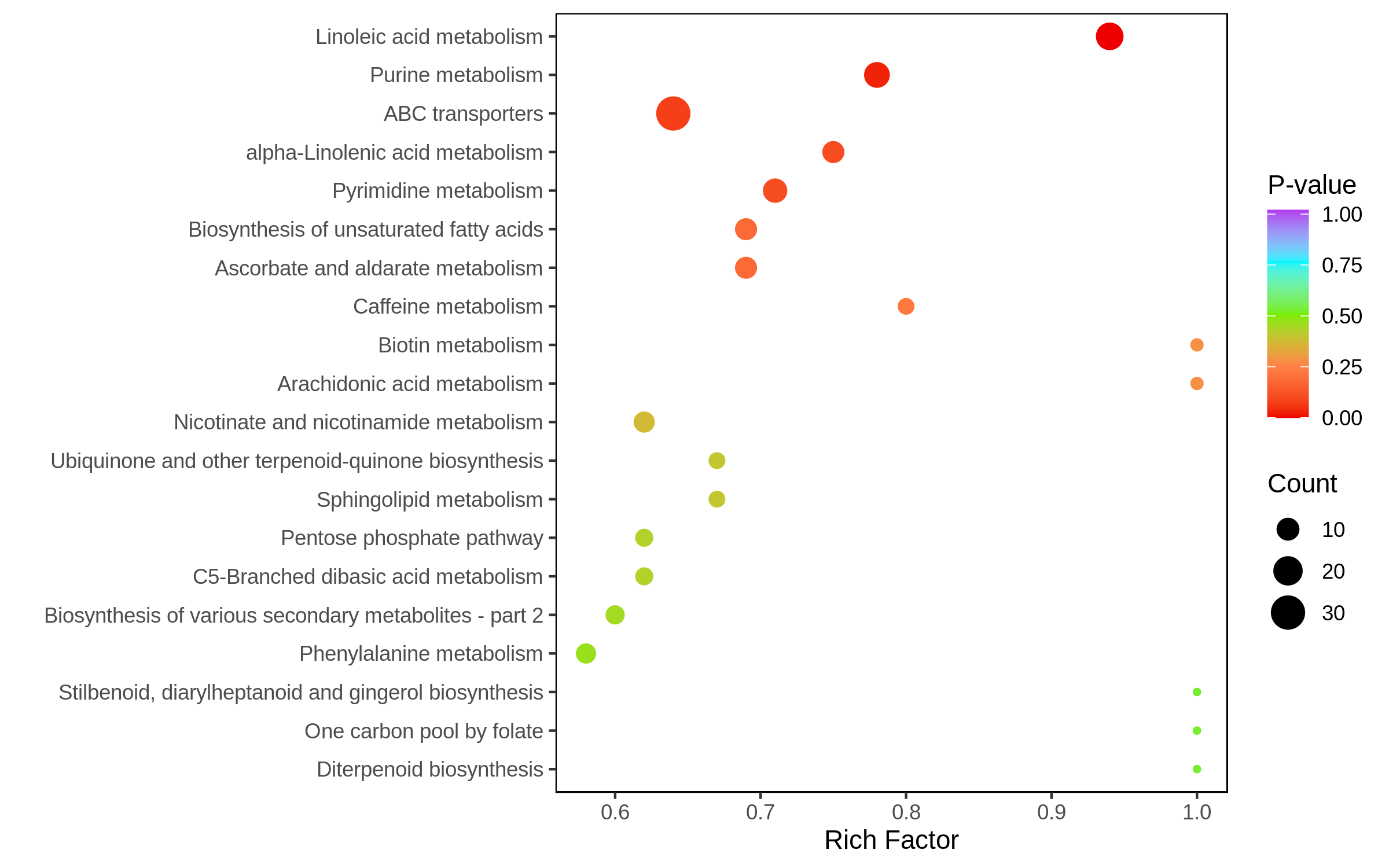

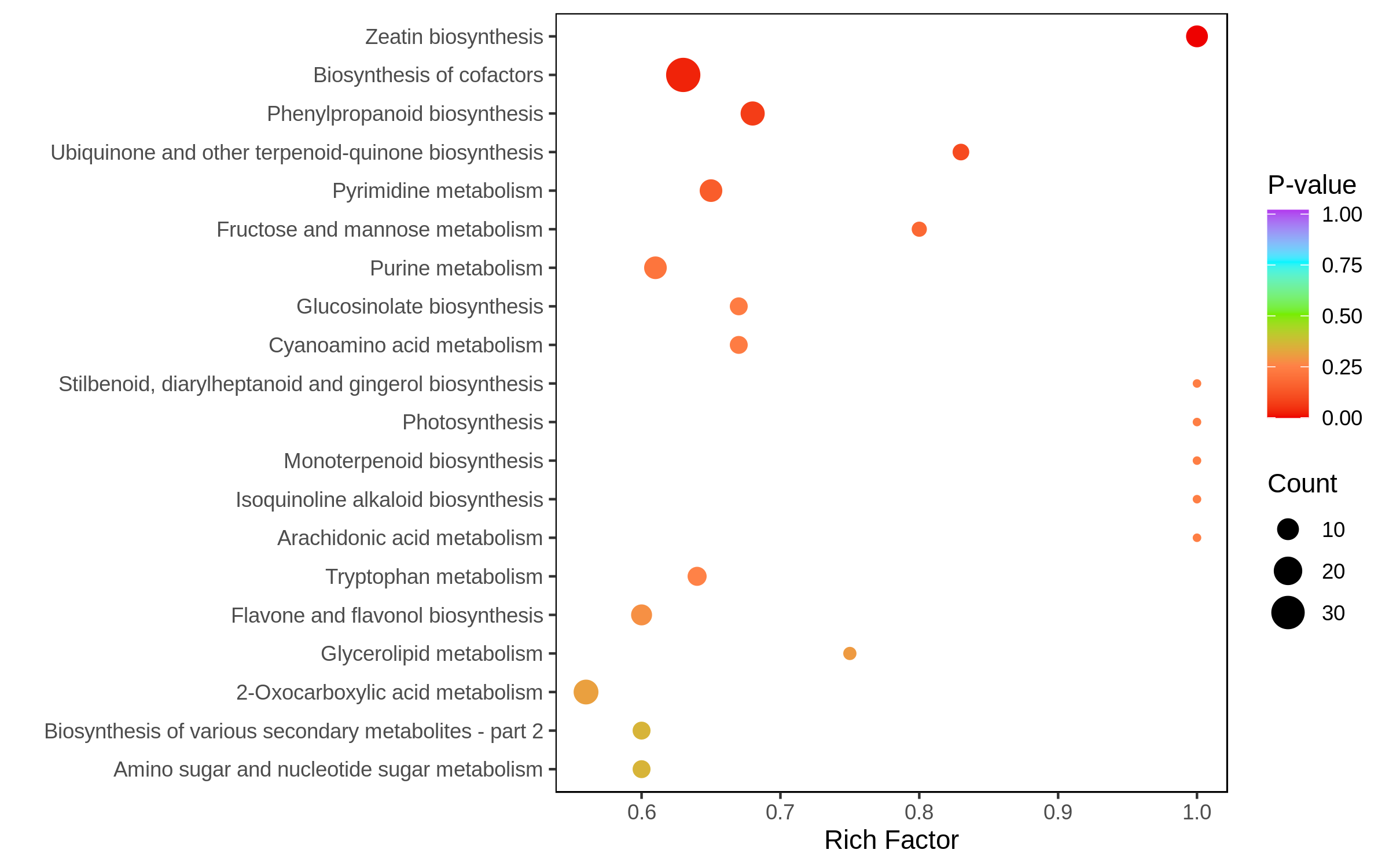
C D


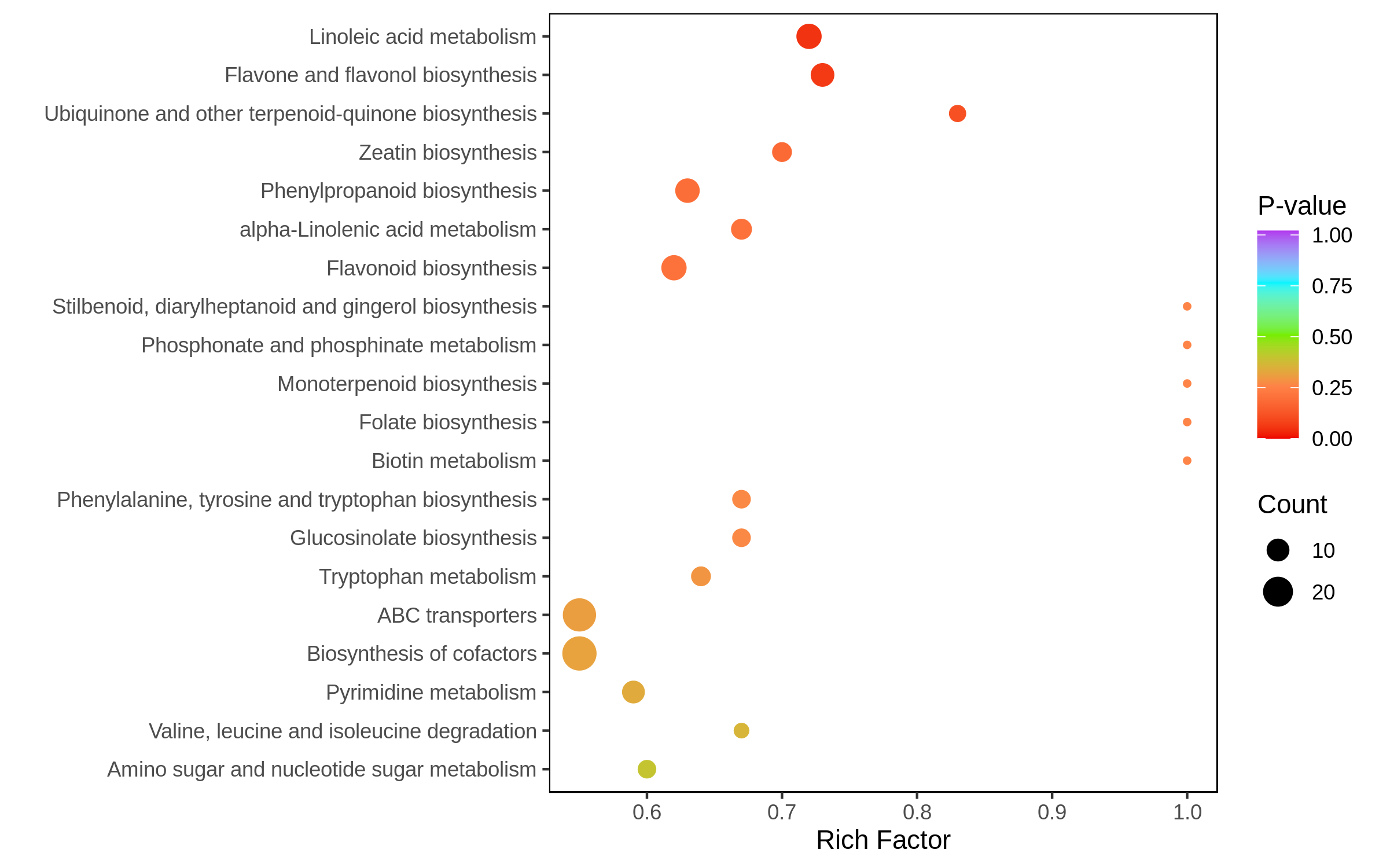

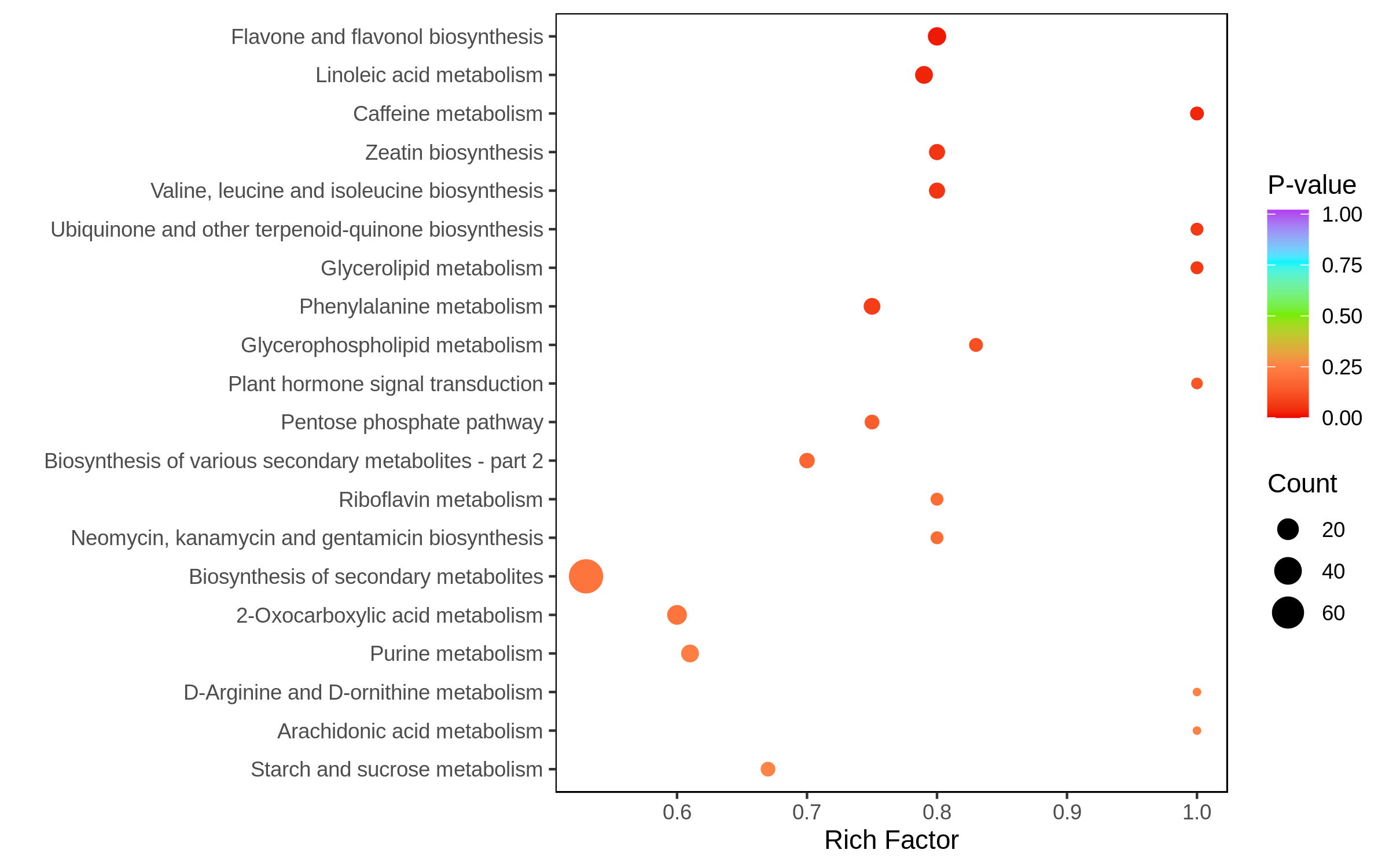
E


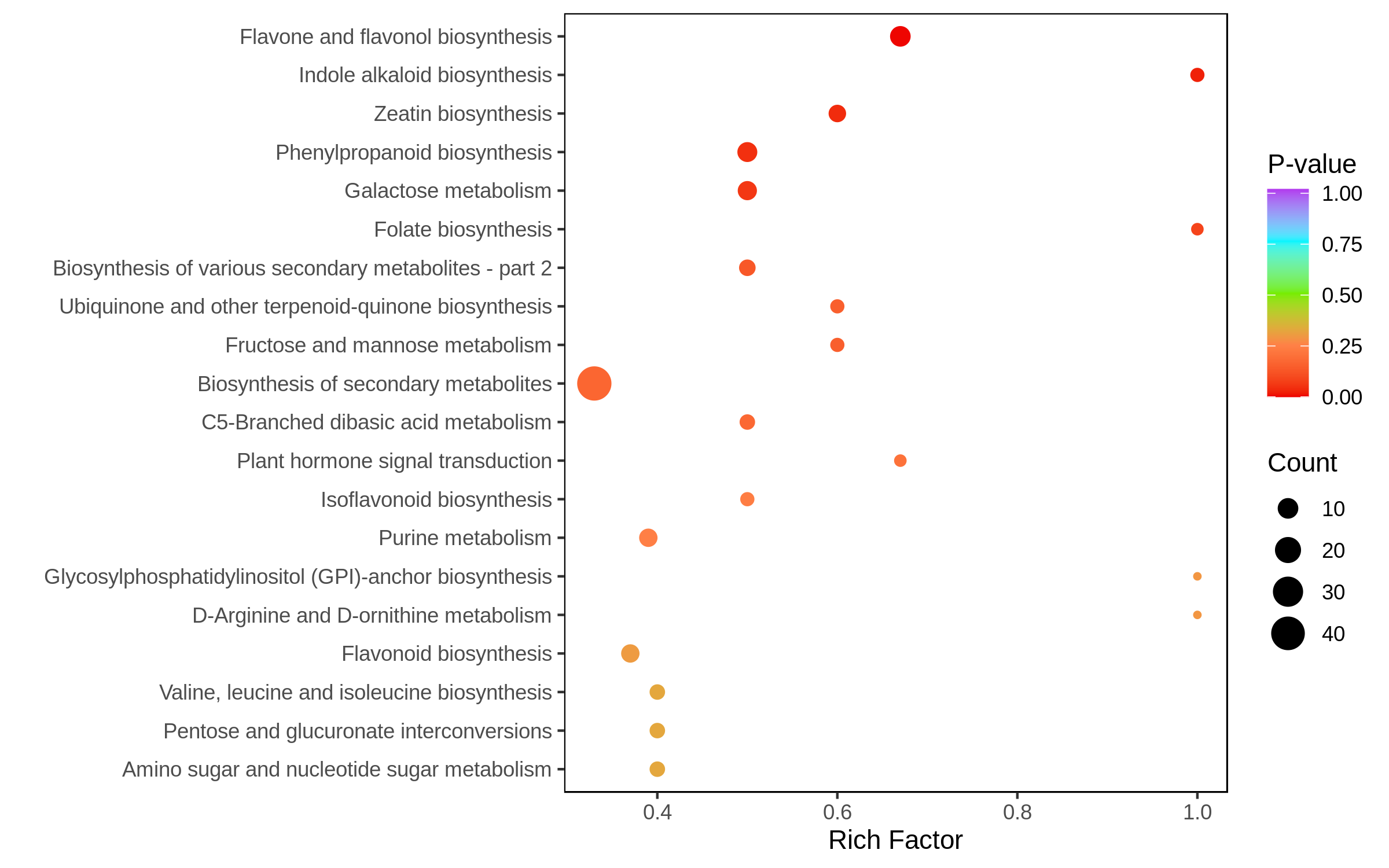


Figure S2 KEGG enrichment analysis showed the potential functions of DAMs in the root vs. rhizome (A), root vs. stem (B), root vs. leaf (C), rhizome vs. stem (D), and stem vs. leaf (E), respectively.


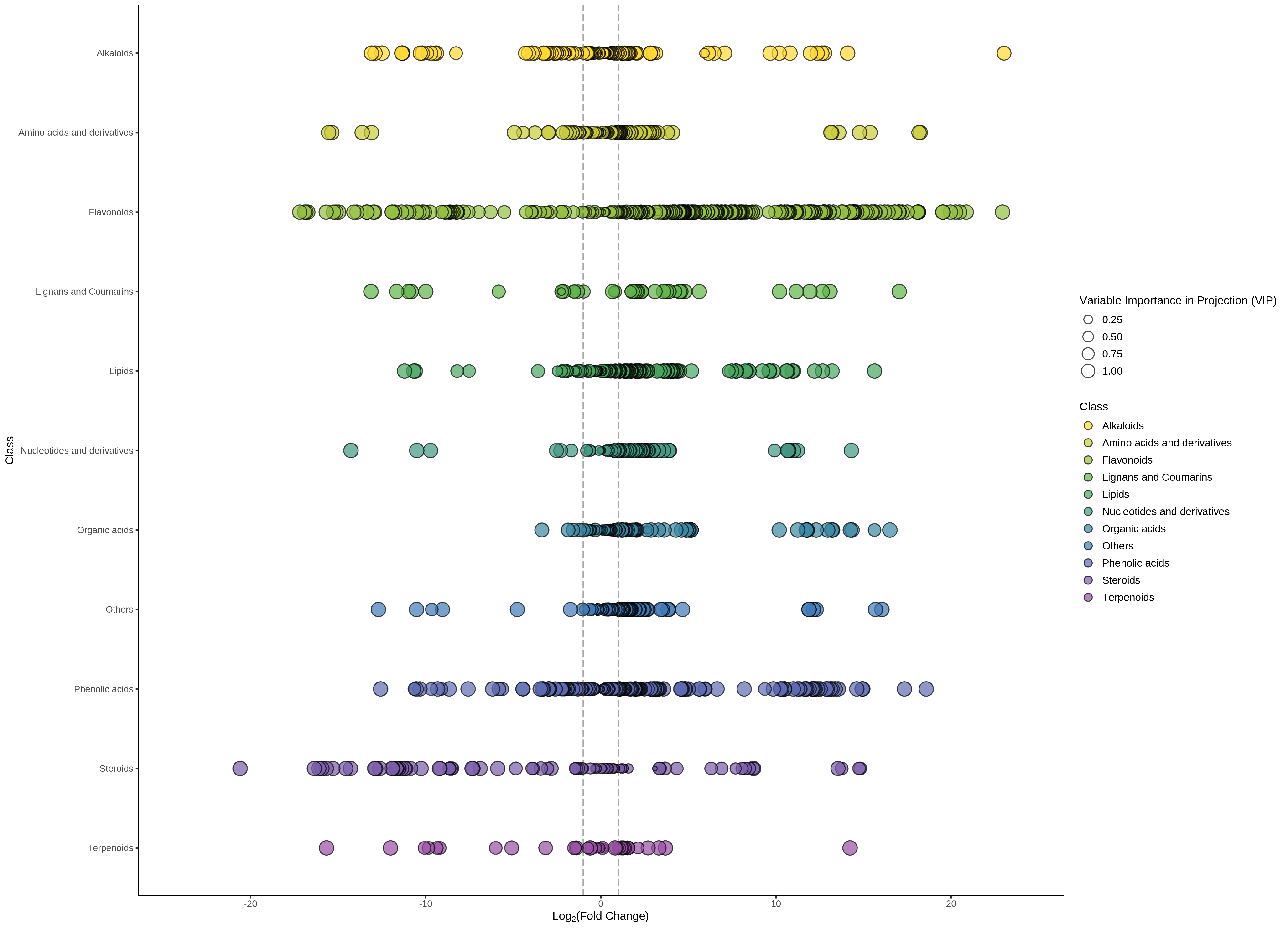
A

B


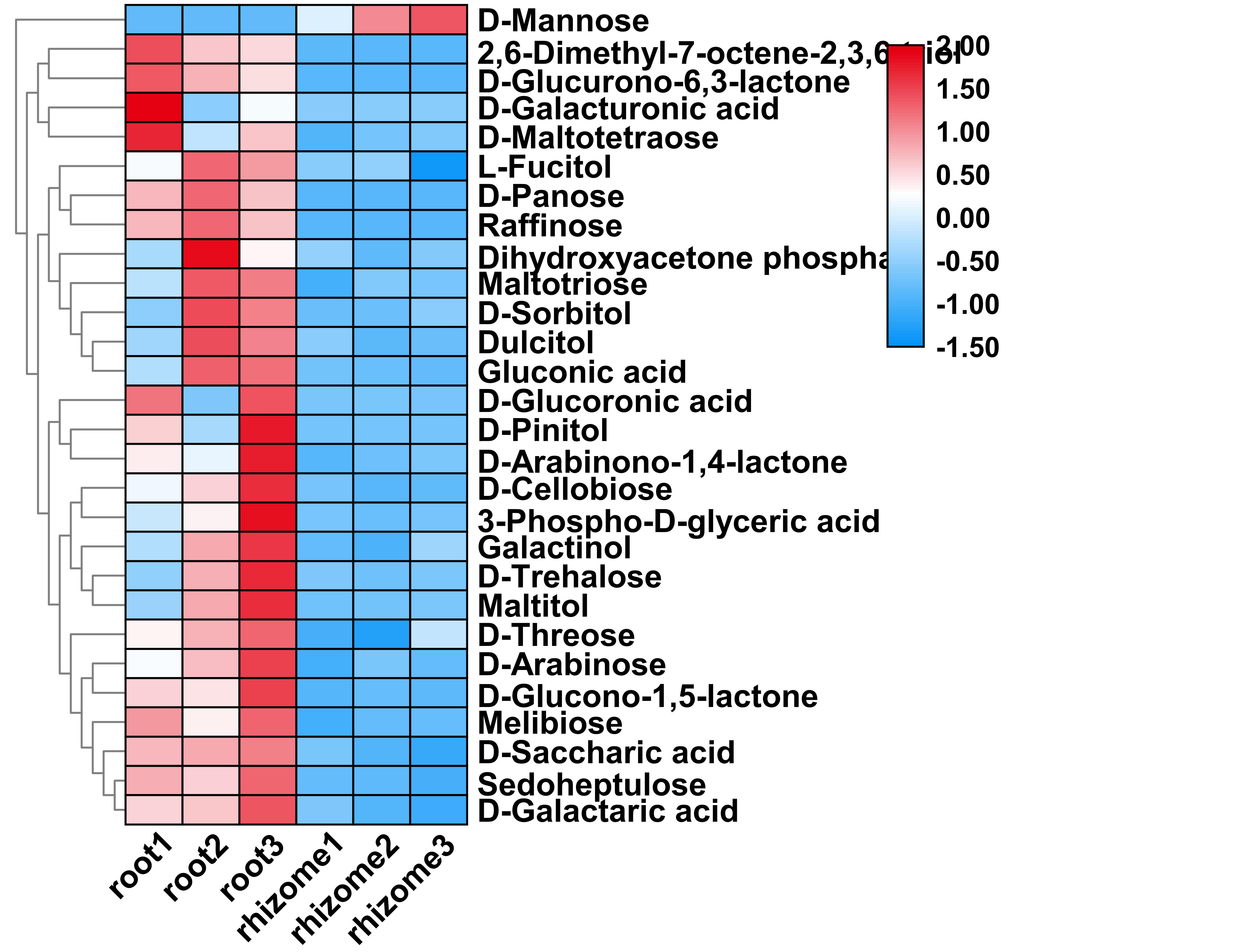


Figure S3 (A) Scatter diagram showed the potential functions of DAMs in leaf vs. Rhizome. (B) Heatmap showing the changes of DAMs involved in saccharides in the root vs rhizome group.

A B


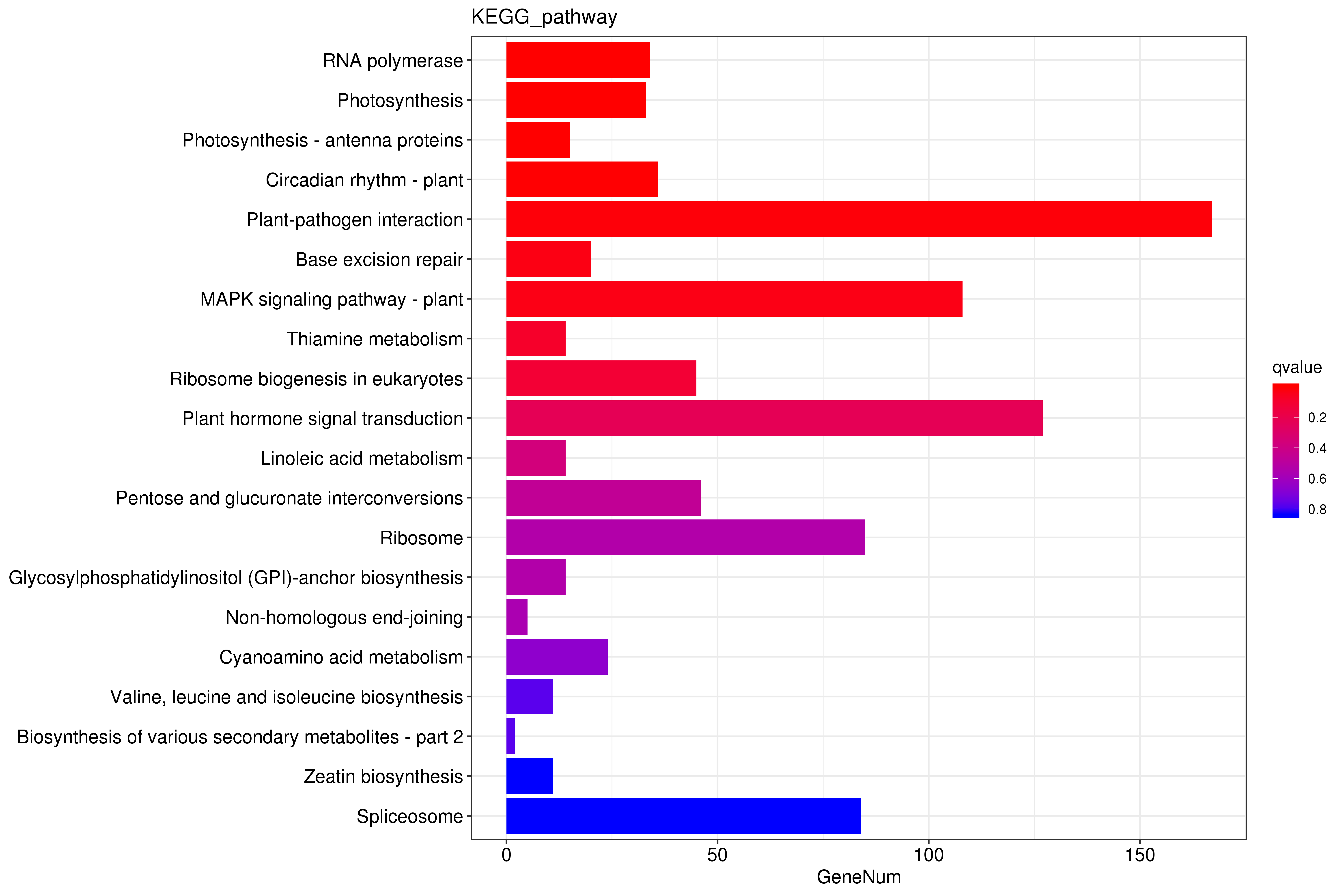

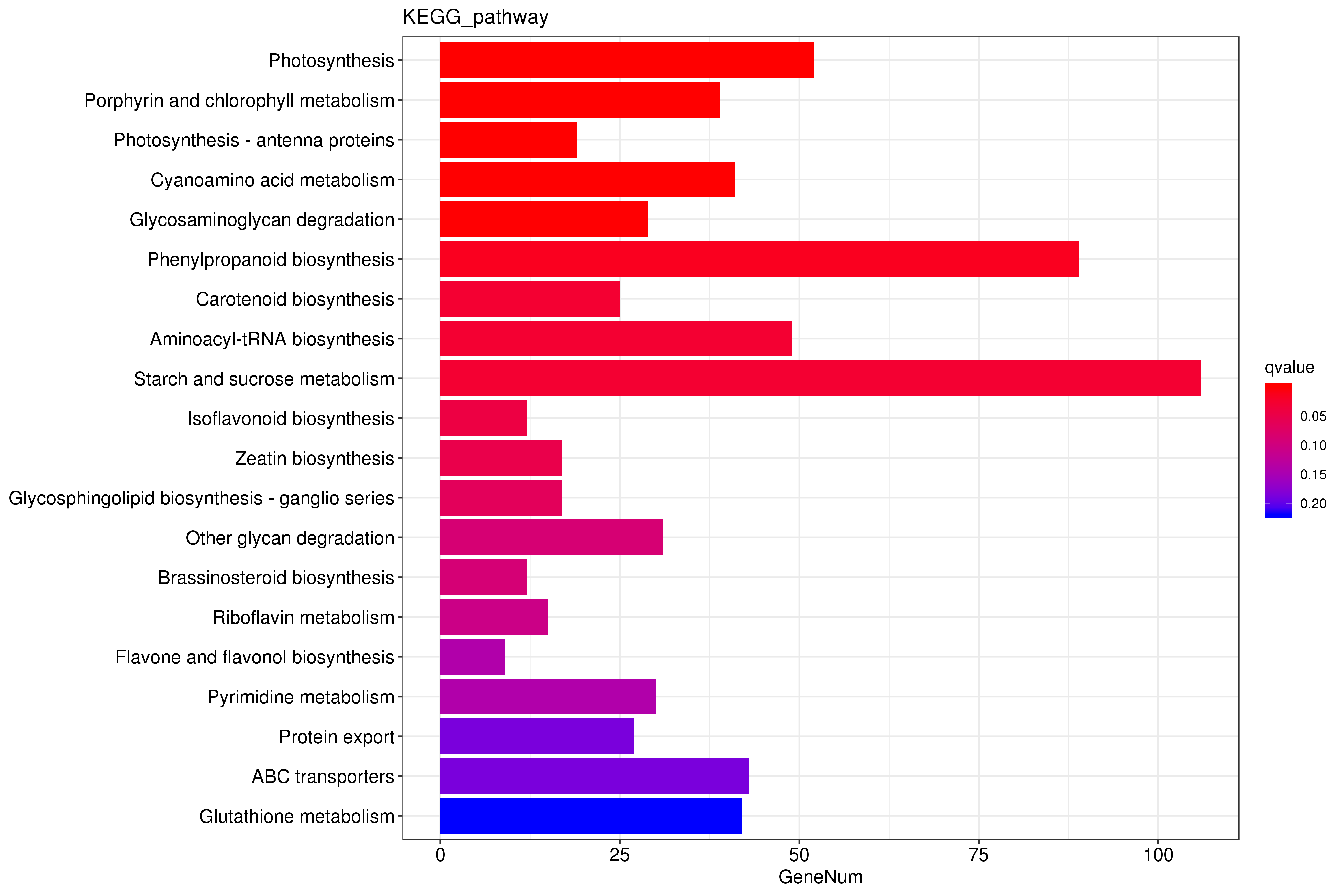
C


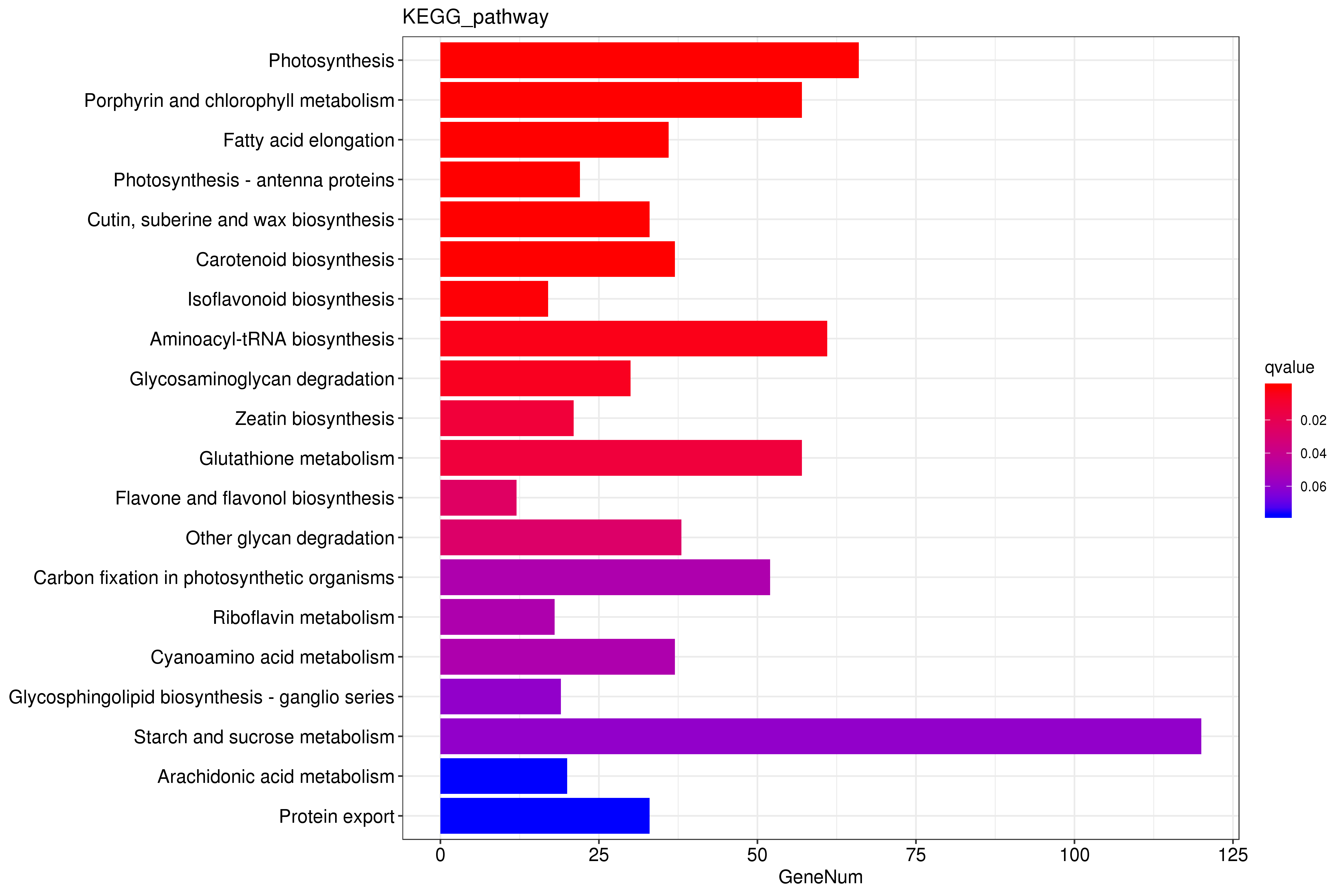


Figure S4 KEGG enrichment analysis showed the potential functions of up-regulated DEGs in the root vs. rhizome (A), rhizome vs. stem (B), and rhizome vs. leaf (C), respectively.


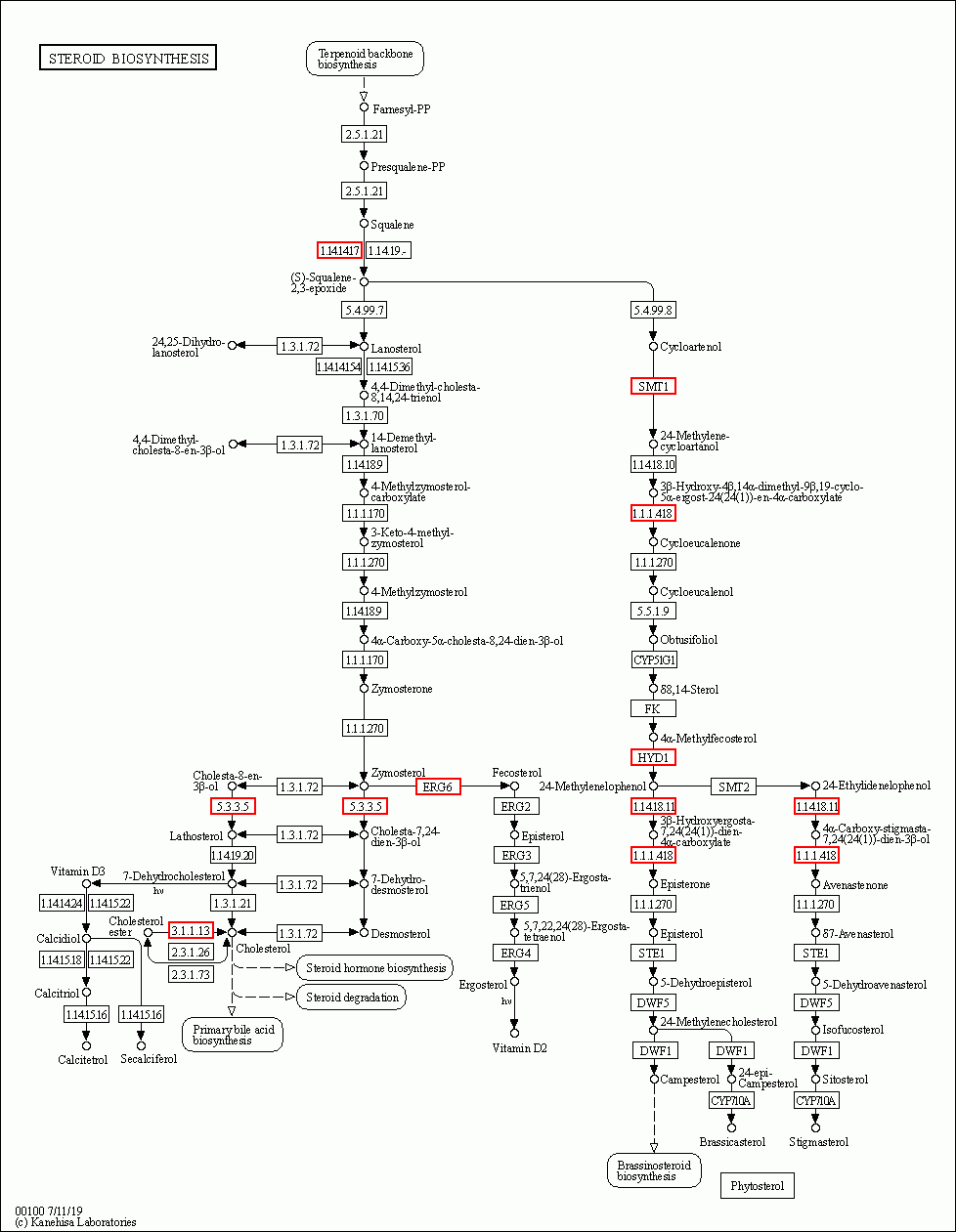


Figure S5 Pathway of steroid biosynthesis in the brown and green modules generated by KEGG. The red boxes represent the annotated genes in steroid biosynthesis (ko00100).


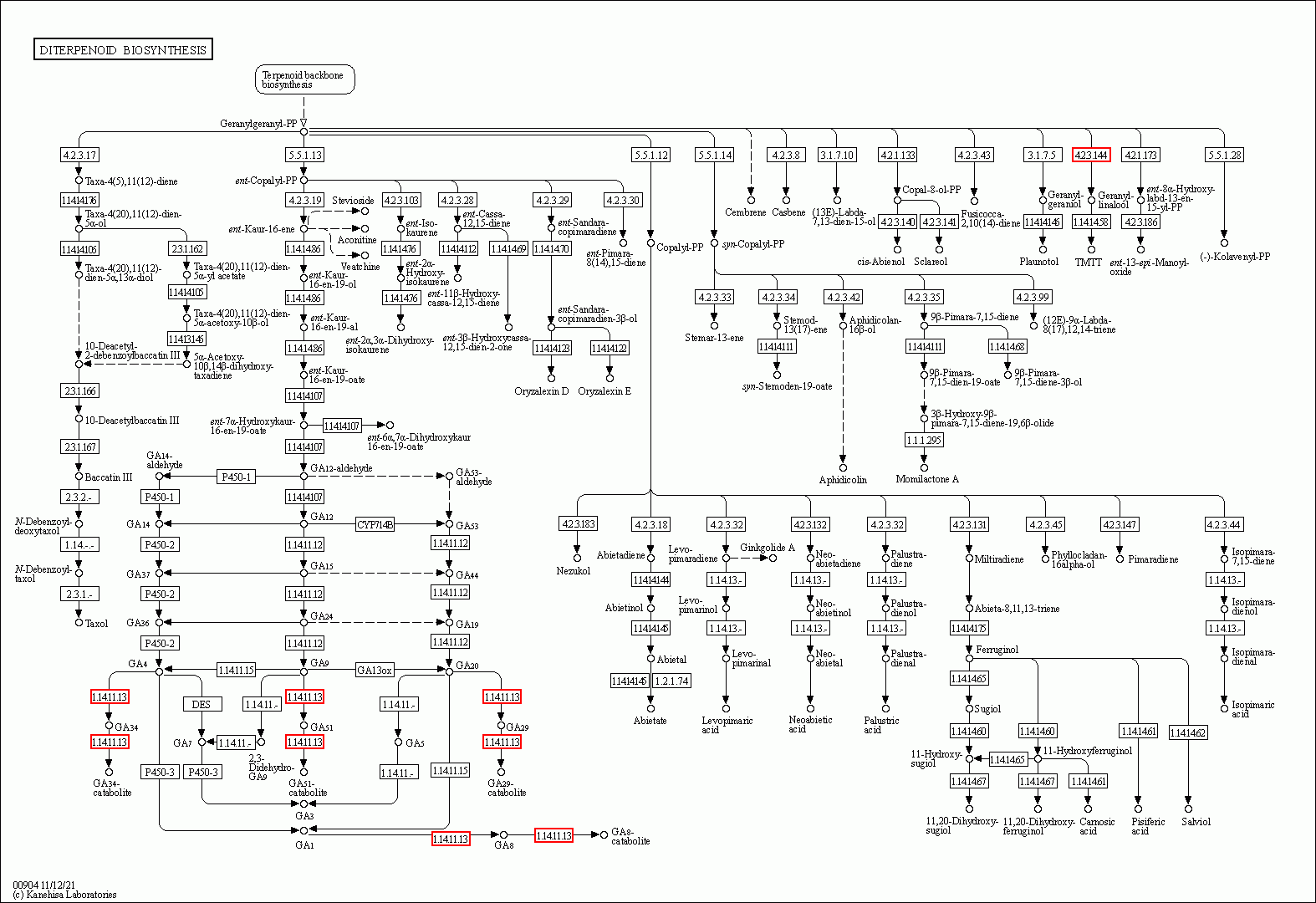


Figure S6 Pathway of diterpenoid biosynthesis in the brown and green modules generated by KEGG. The red boxes represent the annotated genes in diterpenoid biosynthesis (ko00904).


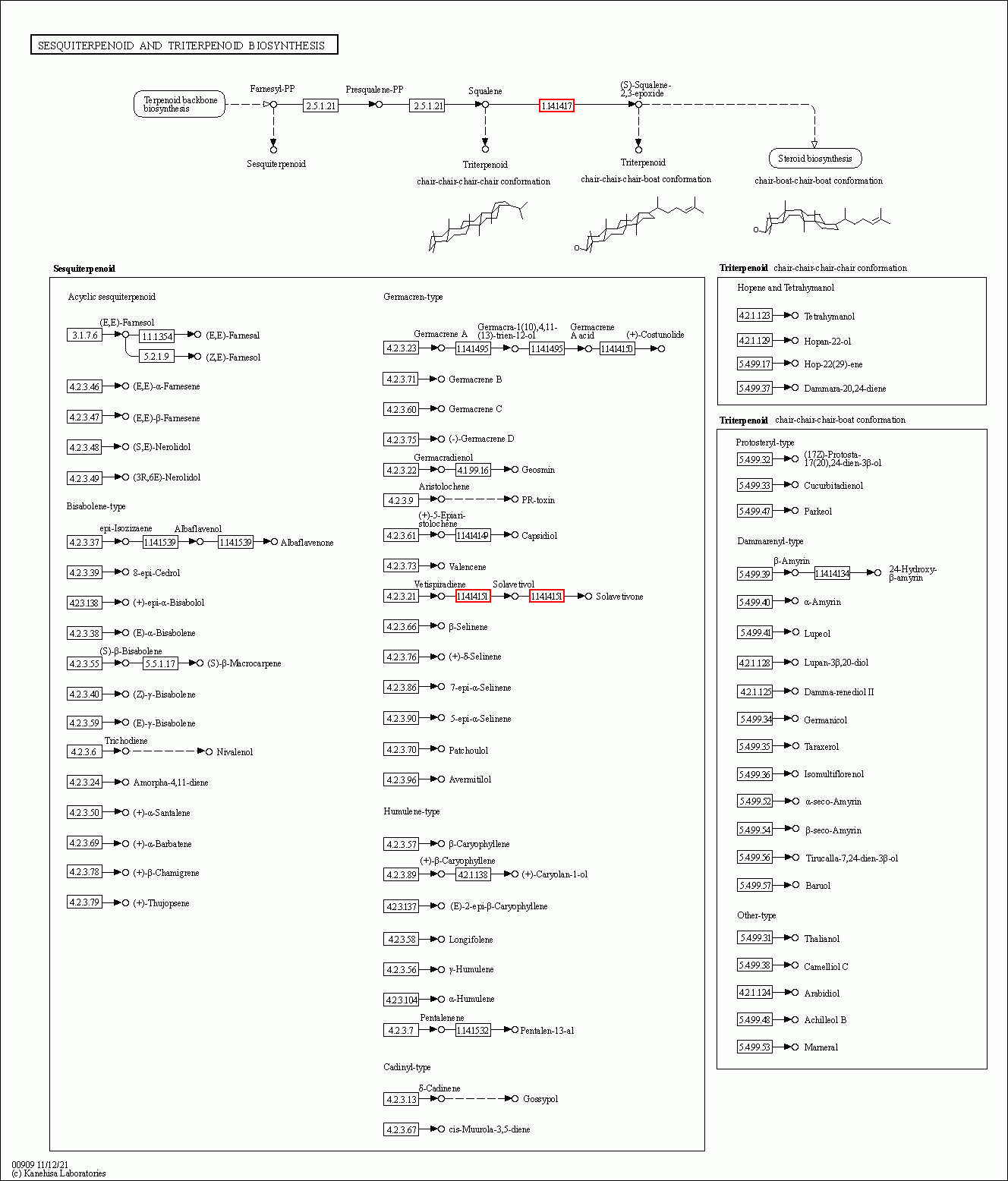


Figure S7 Pathway of sesquiterpenoid and triterpenoid biosynthesis in the brown and green modules generated by KEGG. The red boxes represent the annotated genes in sesquiterpenoid and triterpenoid biosynthesis (ko00909).

A B


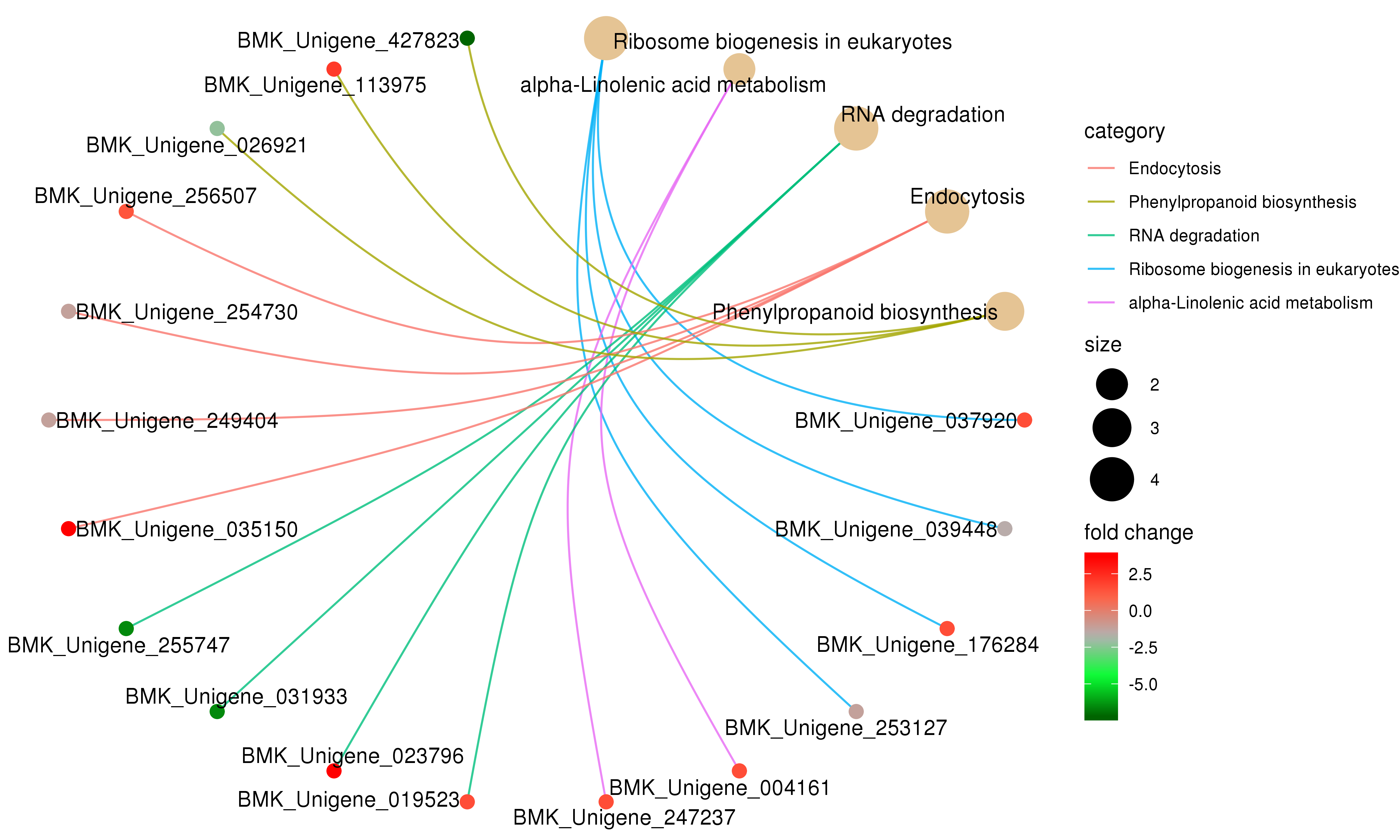

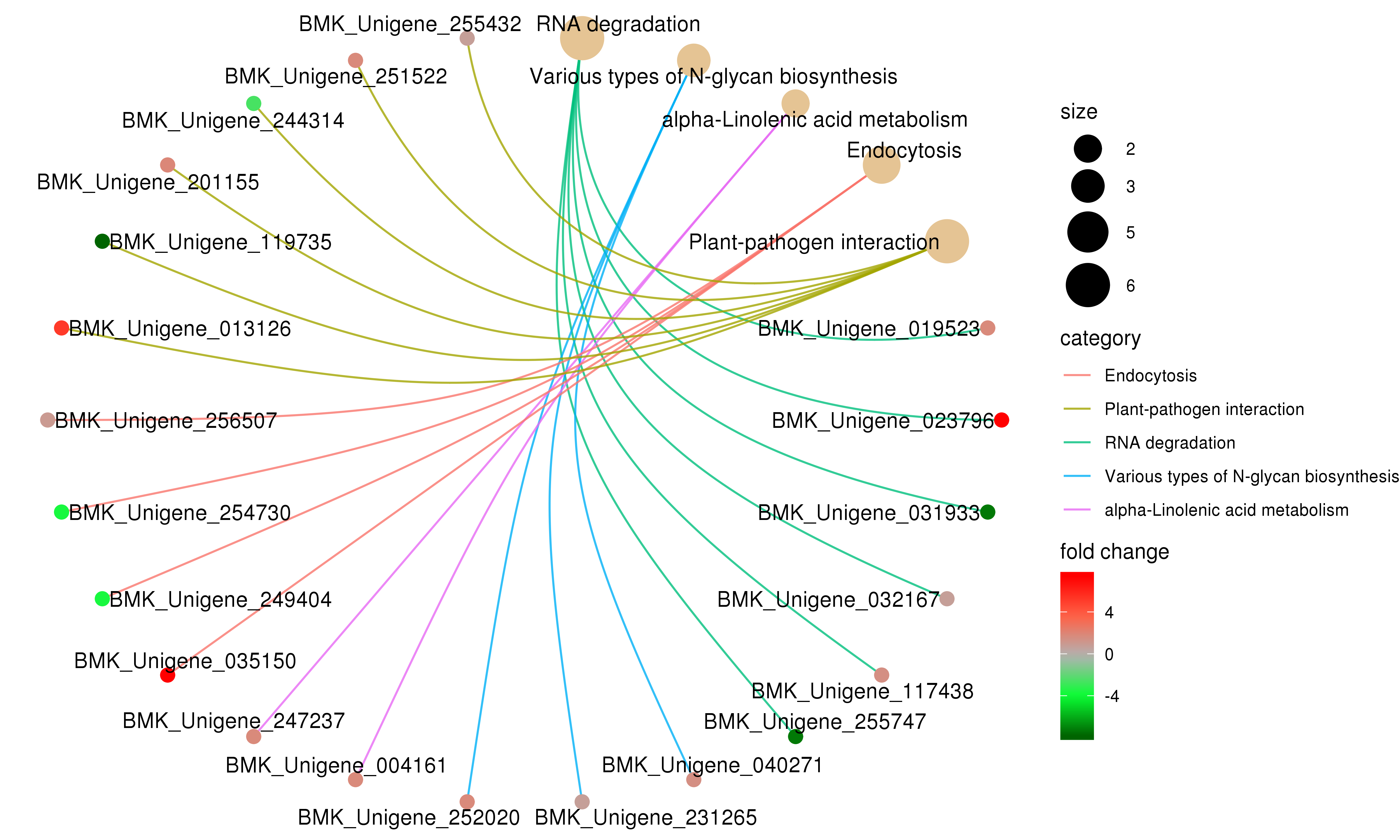
C D


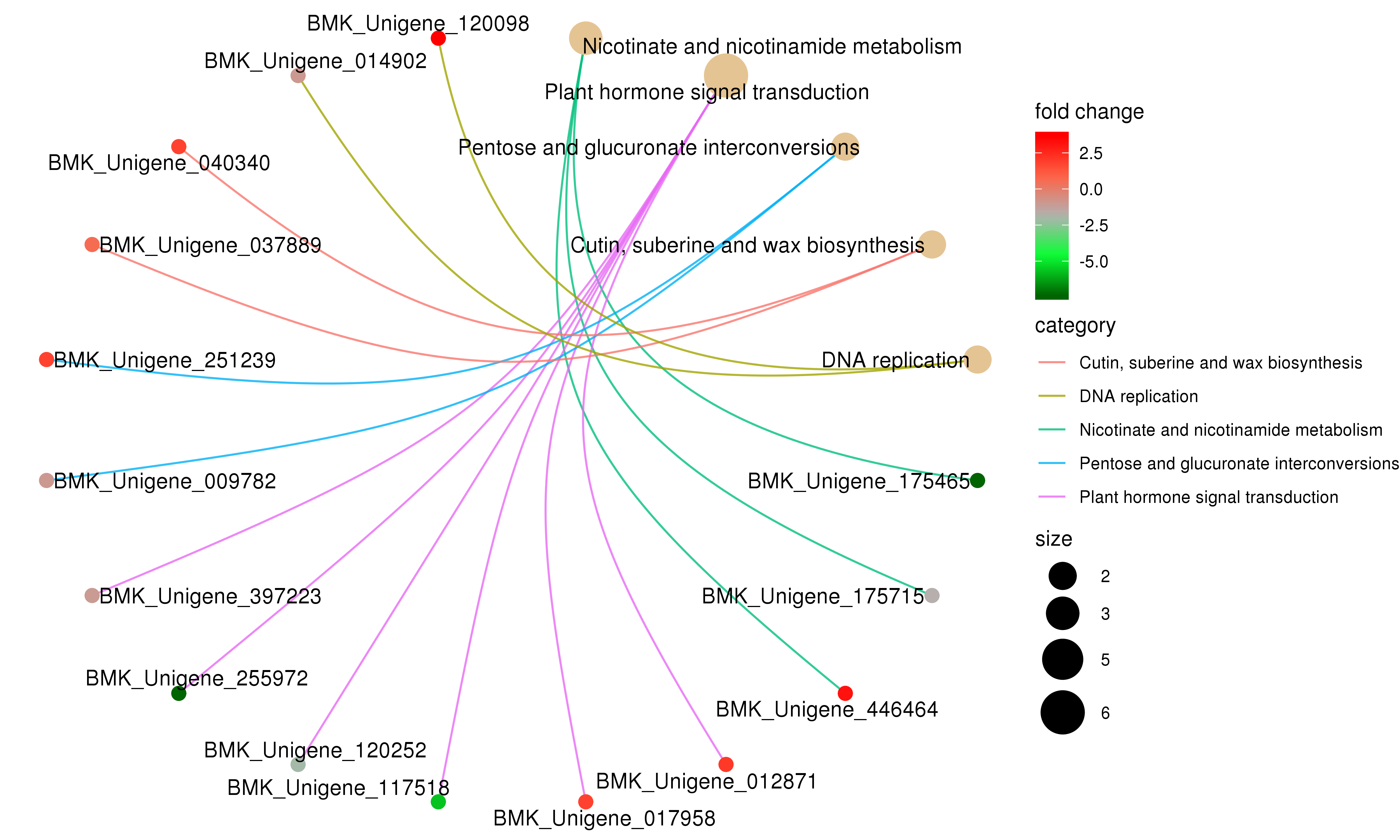

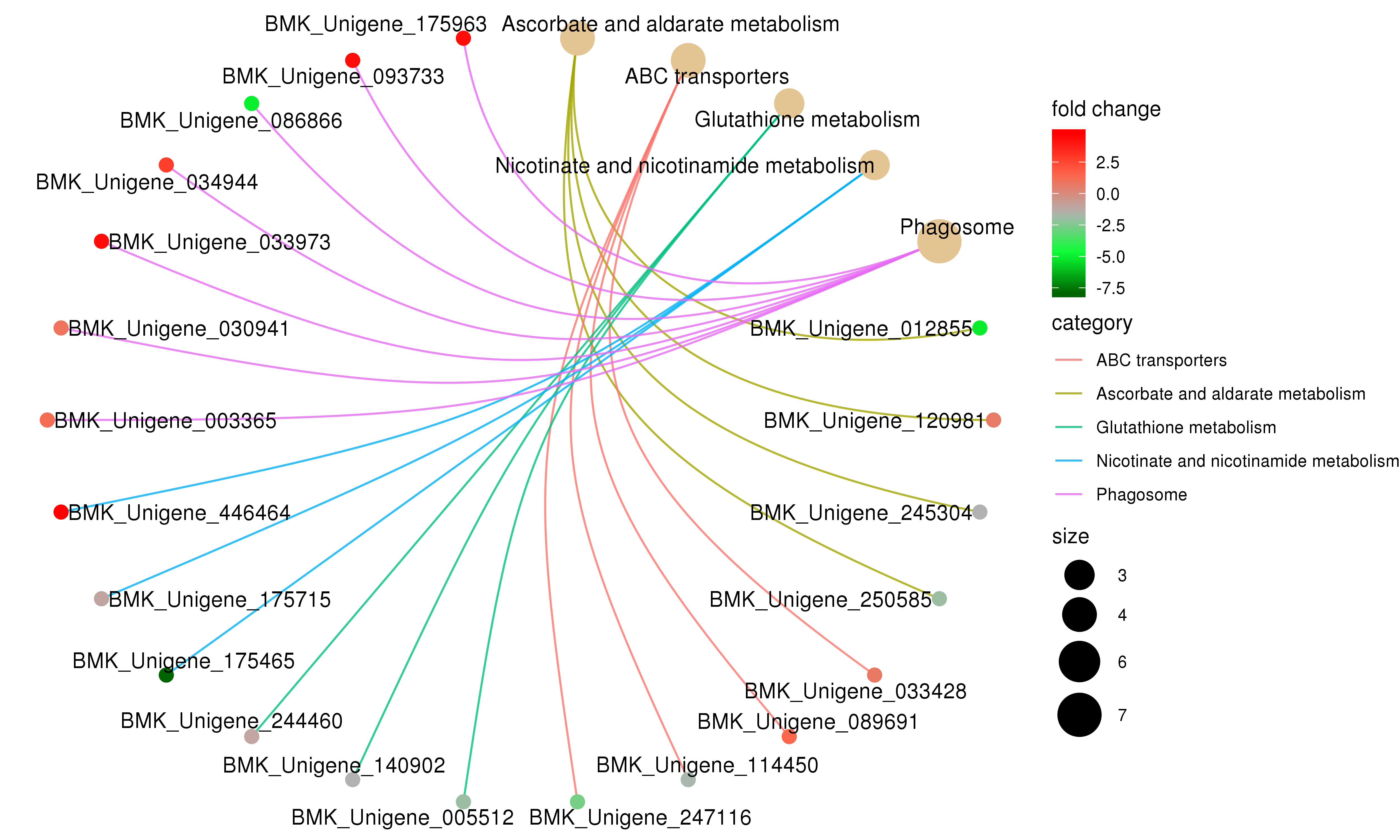
E


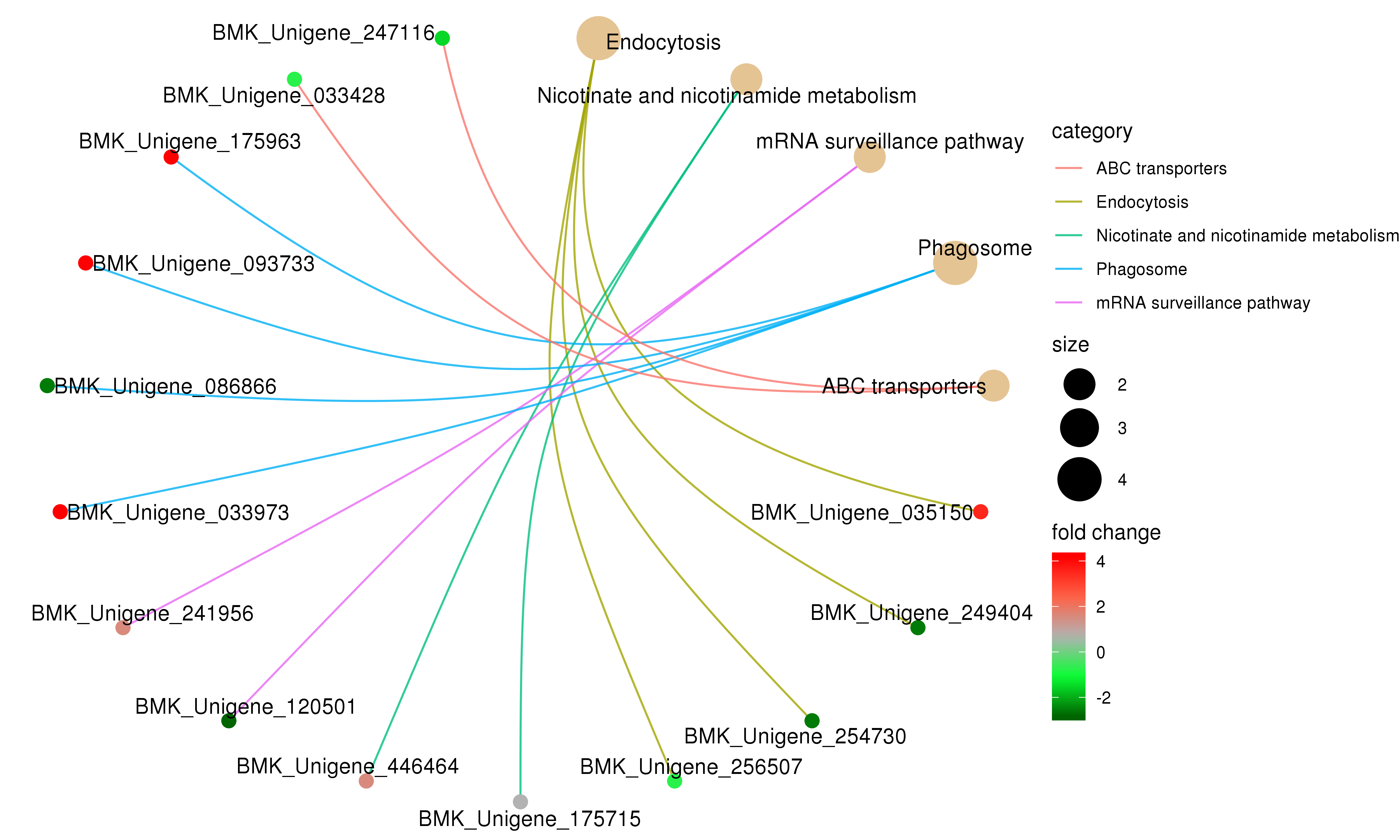


Figure S8 KEGG enrichment analysis showed the potential functions of miRNAs targets in the root vs. stem (A), root vs. leaf (B), rhizome vs. stem (C), rhizome vs. leaf (D), and stem vs. leaf (E), respectively.

A B


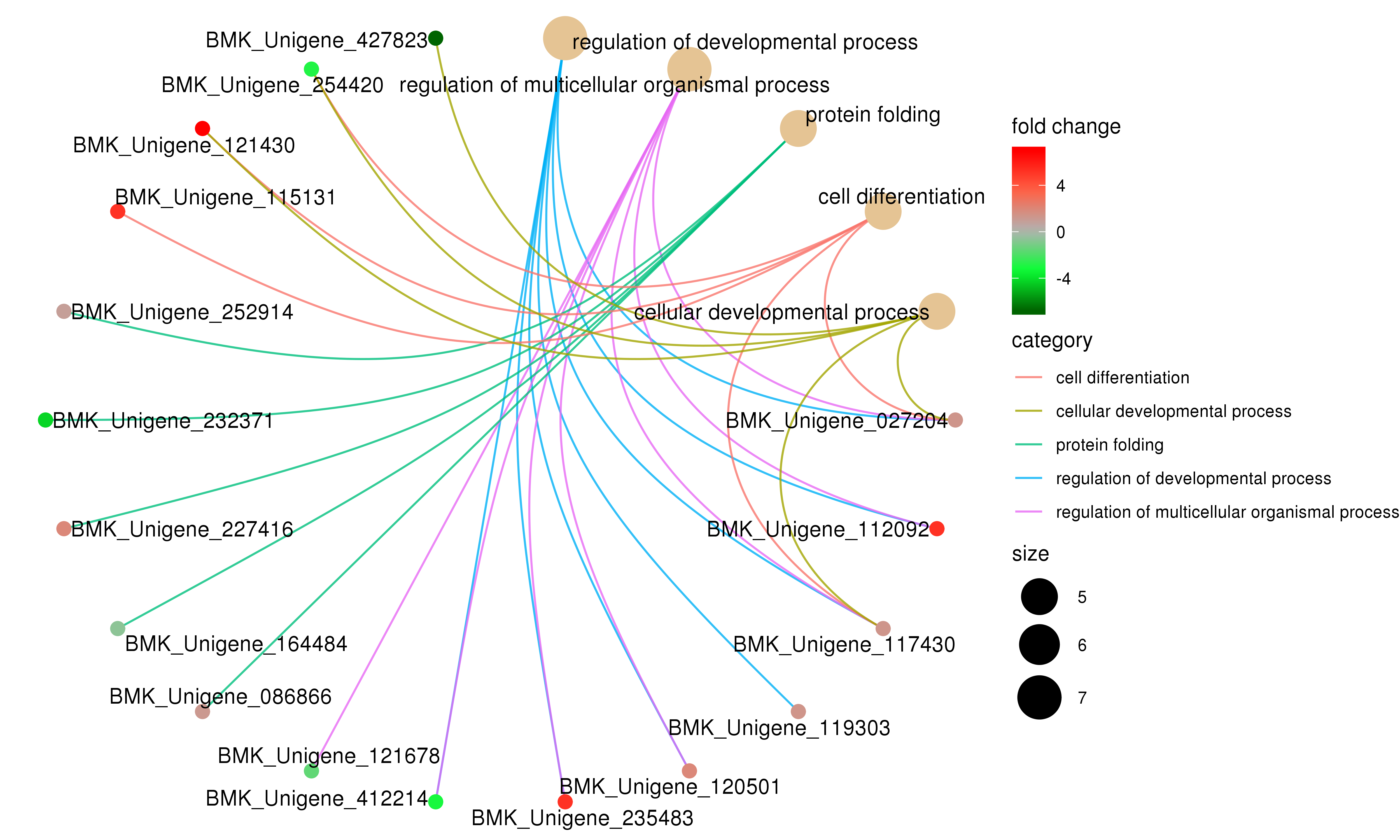

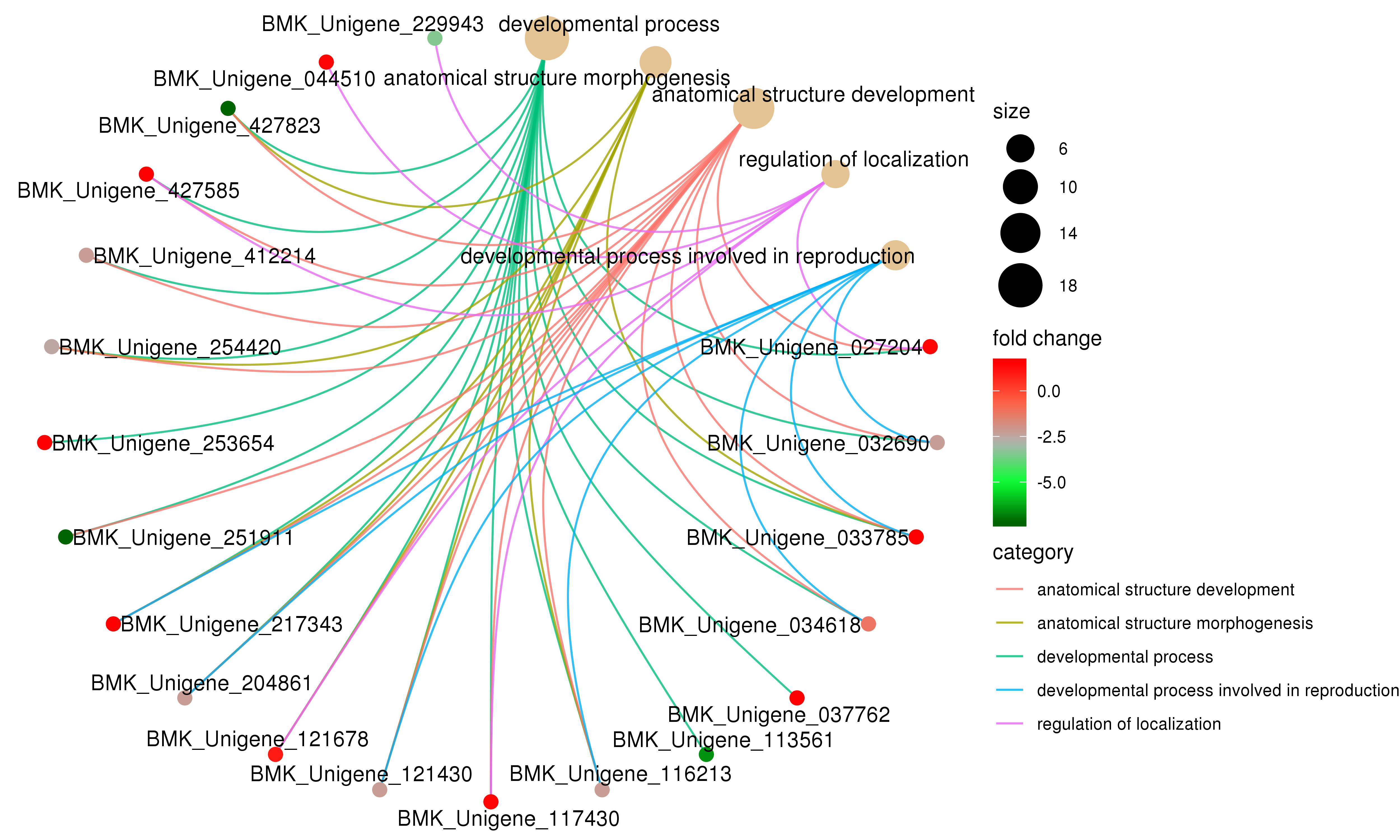

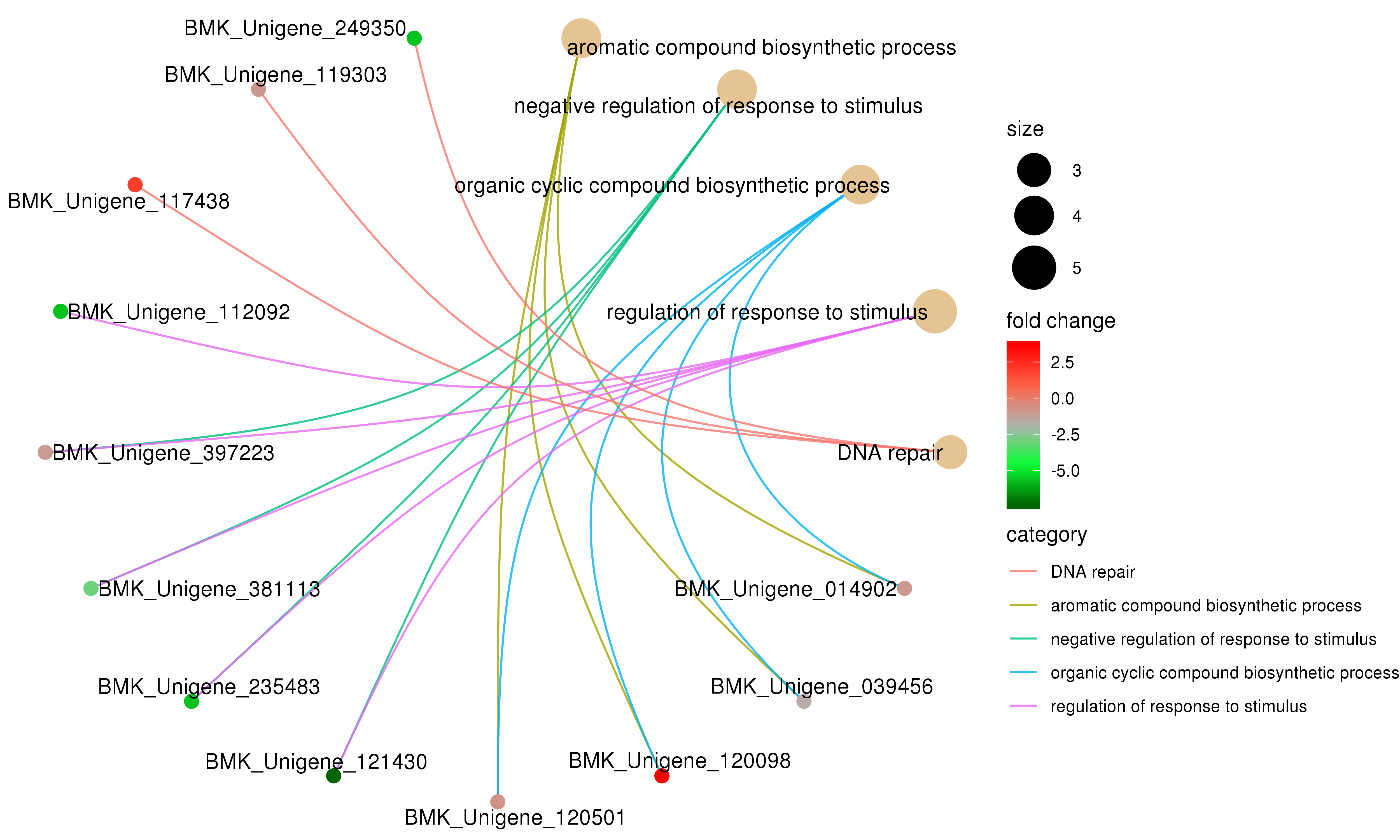

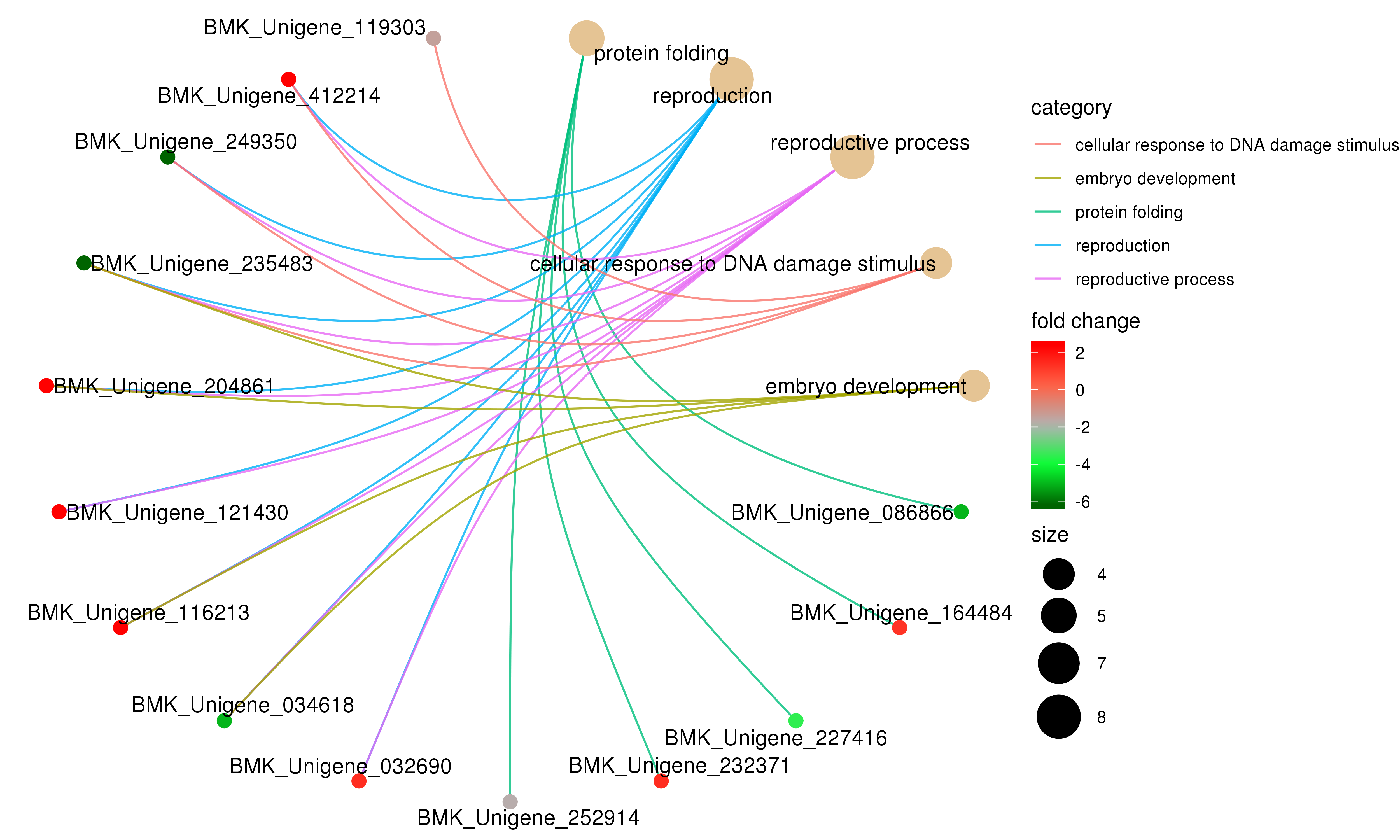
C D

E


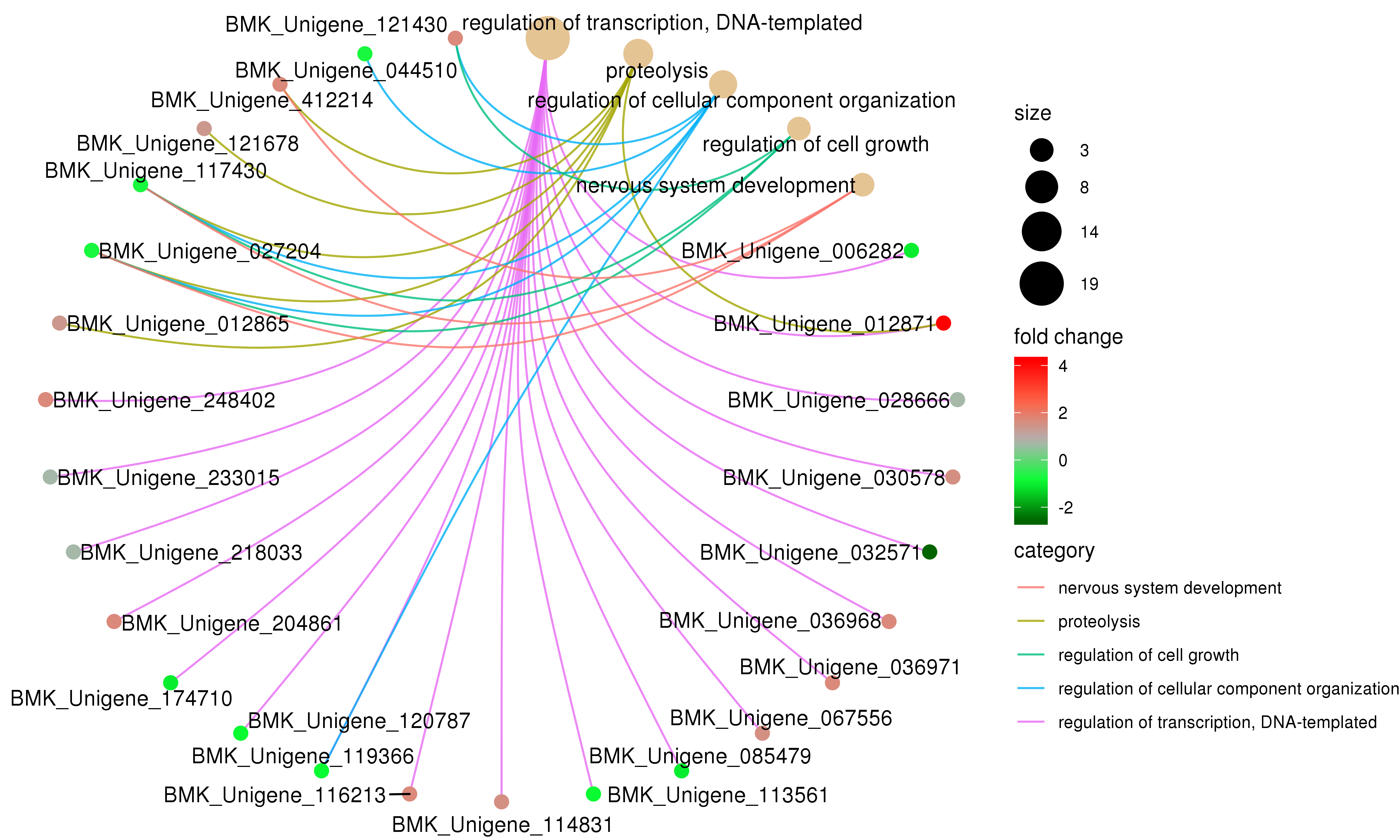


Figure S9 GO enrichment analysis showed the potential functions of miRNAs targets in the root vs. rhizome (A), root vs. stem (B), rhizome vs. stem (C), rhizome vs. leaf (D), and stem vs. leaf (E), respectively.


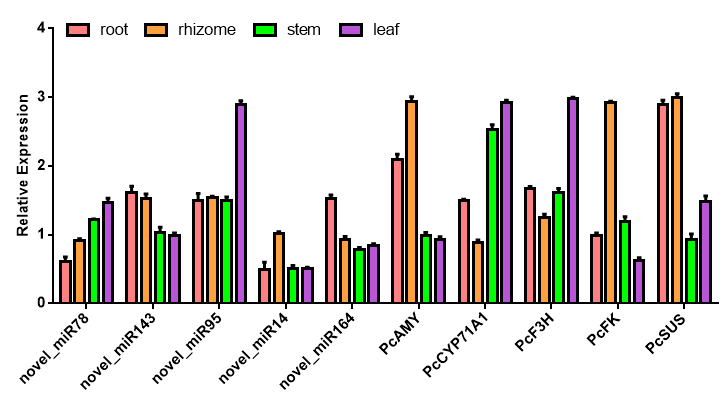
Figure S10 Real-time quantitative RT-qPCR confirmation of *PcFK*, novel_miR95, *PcSUS*, novel_miR143, *PcF3H*, aof_miR164, *PcAMY*, novel_miR14, *PcCYP71A1*, and novel_miR78. Relative gene expressions were analyzed using the 2^−ΔΔCt^ method. Experiments were performed in triplicate. Error bars indicate standard deviation.
