# Supplementary material for: Revealing the mechanisms of the bioactive ingredients accumulation in Polygonatum cyrtonema by multiomics analyses
Source: Front Plant Sci. 2022 Nov 16;13:1055721. doi: 10.3389/fpls.2022.1055721 (PMC9709641; doi:10.3389/fpls.2022.1055721)
Supplement: Supplementary file 2 [file DataSheet_2.doc]

**Supplementary Tables**

Table S1 Primers for genes validated by quantitative real-time PCR (qRT-PCR).

| Genes | Sequences 5’-3’ |
| --- | --- |
| GDAPH-F | CCGTTCATCACCGTTGACTACA |
| GDAPH-R | AACAGTCACAGGCTTTTCACCAA |
| PcFK-F | AACTCATGTAGTTAAGAGGCTAATGATCTTGTT |
| PcFK-R | ATGAAGCTCCATCATTTTGTGTTCTTG |
| PcSUS-F | CACATATTGAGAACATAAAACTCATTTTTTTTTAC |
| PcSUS-R | AAAAAGCTGAGATTCTTTTCACCTCTTTC |
| PcF3H-F | GTCACTTTGATATCTTGAATATAAGACAGTAACTCA |
| PcF3H-R | CTCTTAGAGCACCCCCAATCGC |
| PcAMY-F | TTTCTTGAGCTTTTGGGCTGAGG |
| PcAMY-R | TTGGGACAAAATTTTGGTCAAAATATG |
| PcCYP71A1-F | TTATTGTTGTAGTGTTCTCAAATGTTATTTTCTG |
| PcCYP71A1-R | ATGCCTGAAATTCTGATGTCCATGAC |
| aof_miR164 | TGGAGAAGCAGGGCACGTGCA |
| novel_miR14 | GAGAGTGACAATGAGATTGAG |
| novel_miR78 | GCGACTATGATCACAGTCCTCACA |
| novel_miR95 | GCGATCATAGTCGCAGTCCTAATA |
| novel_miR143 | CAGTTCAAGAAAGCCGTGGAA |
| U6 | CGATACAGAGAAGATTAGCATGG |

Table S2 Statistics of DAMs by pairwise comparison.

| group name | All sig diff | down regulated | up regulated |
| --- | --- | --- | --- |
| Rhizome vs. leaf | 703 | 176 | 527 |
| Rhizome vs. stem | 652 | 214 | 438 |
| Root vs. leaf | 786 | 478 | 308 |
| Stem vs. leaf | 480 | 162 | 318 |
| Root vs. stem | 769 | 522 | 247 |
| Root vs. rhizome | 630 | 557 | 73 |

Table S3 Assessment of the completeness of the *de novo* assembled transcripts by BUSCO

| Type | Number | Percent (%) |
| --- | --- | --- |
| Complete BUSCOs (C) | 402 | 94.6 |
| Complete and single-copy BUSCOs (S) | 235 | 55.3 |
| Complete and duplicated BUSCOs (D) | 167 | 39.3 |
| Fragmented BUSCOs (F) | 11 | 2.6 |
| Missing BUSCOs (M) | 12 | 2.8 |
| Total BUSCO groups searched | 425 | 100 |

Table S4 Statistics of functional annotation of unigenes.

| Anno_Database | Annotated_Number | 300<=length<1000 | length>=1000 |
| --- | --- | --- | --- |
| COG_Annotation | 14003 | 1728 | 12275 |
| GO_Annotation | 35556 | 6532 | 29020 |
| KEGG_Annotation | 29749 | 4907 | 24842 |
| KOG_Annotation | 24507 | 3964 | 20543 |
| Pfam_Annotation | 33801 | 5084 | 28717 |
| Swissprot_Annotation | 29597 | 5293 | 24304 |
| TrEMBL_Annotation | 42042 | 8047 | 33995 |
| eggNOG_Annotation | 36097 | 6560 | 29537 |
| nr_Annotation | 42831 | 8203 | 34628 |
| All_Annotated | 43189 | 8333 | 34852 |

Table S5 Statistics of DEGs by pairwise comparison.

| DEG_Set | All_DEG | up-regulated | down-regulated |
| --- | --- | --- | --- |
| Rhizome vs. leaf | 14747 | 7976 | 6771 |
| Rhizome vs. stem | 13985 | 7136 | 6849 |
| Root vs. leaf | 19291 | 7823 | 11468 |
| Root vs. rhizome | 19383 | 7057 | 12326 |
| Root vs. stem | 17999 | 6471 | 11528 |
| Stem vs. leaf | 8994 | 5394 | 3600 |

Table S6 Statistics of predicted TFs in blue module.

| Gene_ID | Member | Gene_ID | Member |
| --- | --- | --- | --- |
| Unigene_011793 | basic region/leucine zipper 60 | Unigene_227001 | WRKY DNA-binding protein 4 |
| Unigene_012692 | NF-X-like 1 | Unigene_252277 | heat shock transcription factor A6B |
| Unigene_016459 | VIRE2-interacting protein 1 | Unigene_252407 | WRKY family protein |
| Unigene_017025 | WRKY 65 | Unigene_252604 | bHLH family protein |
| Unigene_018208 | GRAS family protein | Unigene_254161 | nuclear factor Y, subunit A10 |
| Unigene_019692 | bZIP family protein | Unigene_254473 | DNA-binding regulator |
| Unigene_022647 | FAR1-related sequence 5 | Unigene_254720 | Trihelix family protein |
| Unigene_025136 | SBP family protein | Unigene_256765 | NAC domain containing protein 28 |
| Unigene_026454 | nuclear factor Y, subunit A10 | Unigene_038138 | auxin response factor 16 |
| Unigene_030476 | SCARECROW-like 8 | Unigene_224735 | auxin response factor 16 |
| Unigene_035009 | WRKY family protein | Unigene_006807 | related to AP2 11 |
| Unigene_035404 | bHLH family protein | Unigene_010958 | ethylene responsive binding factor 3 |
| Unigene_036967 | bZIP family protein | Unigene_112628 | ERF family protein |
| Unigene_037917 | nuclear factor Y, subunit A2 | Unigene_115700 | ethylene responsive binding factor 5 |
| Unigene_040163 | NAC 014 | Unigene_118994 | ERF family protein |
| Unigene_040375 | Trihelix family protein | Unigene_174725 | ethylene responsive binding factor 3 |
| Unigene_043792 | basic region/leucine zipper 16 | Unigene_188789 | ethylene responsive binding factor 1 |
| Unigene_045292 | squamosa promoter 14 | Unigene_224195 | related to AP2 9 |
| Unigene_086338 | heat shock factor 4 | Unigene_246687 | erf domain protein 9 |
| Unigene_110605 | sequence-specific factors | Unigene_250825 | related to AP2 4 |
| Unigene_114111 | basic pentacysteine 6 | Unigene_117510 | light-regulated zinc finger protein 1 |
| Unigene_114375 | WRKY40 | Unigene_176446 | light-regulated zinc finger protein 1 |
| Unigene_116034 | bZIP family protein | Unigene_254518 | B-box type zinc finger protein |
| Unigene_116973 | NAC family protein | Unigene_039536 | response regulator 10 |
| Unigene_117764 | WRKYprotein 28 | Unigene_016963 | G2-like family protein |
| Unigene_117859 | bHLH family protein | Unigene_112737 | G2-like family protein |
| Unigene_118080 | basic pentacysteine 6 | Unigene_115709 | G2-like family protein |
| Unigene_119041 | SCARECROW-like 14 | Unigene_192199 | G2-like family protein |
| Unigene_119750 | WRKY family protein | Unigene_198107 | G2-like family protein |
| Unigene_119780 | nuclear factor Y, subunit A10 | Unigene_208797 | phosphate starvation response 1 |
| Unigene_120102 | bHLH family protein | Unigene_226090 | G2-like family protein |
| Unigene_120618 | FAR1-related sequence 5 | Unigene_176174 | growth-regulating factor 4 |
| Unigene_120709 | signal responsive 1 | Unigene_211889 | growth-regulating factor 1 |
| Unigene_121244 | nuclear factor Y, subunit C2 | Unigene_039657 | TALE family protein |
| Unigene_163425 | NIN like protein 7 | Unigene_218357 | TALE family protein |
| Unigene_164686 | bHLH family protein | Unigene_120274 | HD-ZIP family protein |
| Unigene_165556 | FAR1-related sequence 11 | Unigene_009903 | relative of early flowering 6 |
| Unigene_165622 | cycling DOF factor 2 | Unigene_017015 | C3H family protein |
| Unigene_165766 | heat shock A1D | Unigene_031231 | indeterminate(ID)-domain 12 |
| Unigene_168833 | WRKYprotein 51 | Unigene_033319 | C3H family protein |
| Unigene_169992 | GRAS family protein | Unigene_052133 | C3H family protein |
| Unigene_171395 | GRAS family protein | Unigene_071785 | zinc finger nuclease 3 |
| Unigene_172015 | nuclear factor Y, subunit C10 | Unigene_090011 | C3H family protein |
| Unigene_175933 | BIG PETAL P | Unigene_115050 | zinc finger protein 7 |
| Unigene_176211 | WRKY protein 35 | Unigene_115146 | zinc-finger protein 2 |
| Unigene_176342 | FAR1-related sequence 5 | Unigene_121556 | CCCH-type with ARM repeat domain |
| Unigene_176449 | bHLH family protein | Unigene_173487 | histone deacetylase 2C |
| Unigene_176671 | nuclear factor Y, subunit A5 | Unigene_175122 | indeterminate(ID)-domain 7 |
| Unigene_176796 | TCP family protein | Unigene_175522 | C2H2 family protein |
| Unigene_183010 | WRKY family protein | Unigene_189806 | C3H family protein |
| Unigene_188534 | NAC 74 | Unigene_216216 | indeterminate(ID)-domain 2 |
| Unigene_194185 | FAR1-related sequence 5 | Unigene_222792 | C3H family protein |
| Unigene_196745 | bHLH family protein | Unigene_244006 | indeterminate(ID)-domain 4 |
| Unigene_198148 | bHLH family protein | Unigene_246329 | zinc-finger protein 2 |
| Unigene_198888 | WRKY protein 3 | Unigene_256755 | CCCH-type with ARM repeat domain |
| Unigene_206697 | WRKY protein 33 | Unigene_032505 | myb domain protein 97 |
| Unigene_207076 | bZIP family protein | Unigene_182574 | MYB-like 102 |
| Unigene_213559 | Trihelix family protein | Unigene_226806 | myb domain protein 33 |
| Unigene_218399 | squamosa promoter 2 | Unigene_256430 | myb domain protein 3r-3 |
| Unigene_220696 | E2F transcription factor 3 | Unigene_112194 | DNA binding |
| Unigene_224337 | NAC57 | Unigene_173029 | myb domain protein 61 |
| Unigene_256592 | TRF-like 6 | Unigene_217903 | DNA -containing protein |

Table S7 Statistics of known and novel miRNAs.

| Sample | Known-miRNAs | Novel-miRNAs | Total |
| --- | --- | --- | --- |
| leaf1 | 16 | 132 | 148 |
| leaf2 | 17 | 139 | 156 |
| leaf3 | 17 | 128 | 145 |
| rhizomel1 | 17 | 122 | 139 |
| rhizomel2 | 15 | 115 | 130 |
| rhizomel3 | 9 | 123 | 132 |
| root1 | 16 | 126 | 142 |
| root2 | 18 | 122 | 140 |
| root3 | 17 | 128 | 145 |
| stem1 | 18 | 136 | 154 |
| stem2 | 16 | 137 | 153 |
| stem3 | 16 | 140 | 156 |
| Total | 20 | 149 | 169 |

Table S8 Statistics of miRNA family.

| Family | Number | Family | Number |
| --- | --- | --- | --- |
| MIR159 | 9 | MIR477 | 5 |
| MIR164 | 6 | MIR5225 | 3 |
| MIR166 | 9 | MIR5291 | 3 |
| MIR167_1 | 10 | MIR6108 | 6 |
| MIR168 | 9 | MIR6140 | 4 |
| MIR172 | 15 | MIR7502 | 5 |
| MIR319 | 5 | MIR7708 | 4 |
| MIR3631 | 6 | MIR774 | 4 |
| MIR395 | 9 | MIR7984 | 4 |
| MIR396 | 8 | MIR827_2 | 5 |
| MIR396_2 | 4 | MIR833 | 3 |
| MIR408_2 | 4 | MIR838 | 4 |
| MIR4227 | 4 | MIR842 | 5 |
| MIR444 | 4 | MIR845_1 | 4 |
| MIR946 | 4 | MIR8762 | 4 |

Table S9 Statistics of DEMs by pairwise comparison.

| DEM Set | DEG Number | up-regulated | down-regulated |
| --- | --- | --- | --- |
| Rhizome vs. leaf | 79 | 39 | 40 |
| Rhizome vs. stem | 57 | 26 | 31 |
| Root vs. leaf | 74 | 40 | 34 |
| Root vs. rhizome | 50 | 25 | 25 |
| Root vs. stem | 62 | 32 | 30 |
| Stem vs. leaf | 50 | 25 | 25 |

Table S10 Statistics of functional annotation of all miRNAs targets.

| DEM Set | Total | COG | GO | KEGG | KOG | NR | Pfam | Swiss-Prot | eggNOG |
| --- | --- | --- | --- | --- | --- | --- | --- | --- | --- |
| Rhizome vs. leaf | 484 | 86 | 311 | 208 | 165 | 453 | 225 | 196 | 264 |
| Rhizome vs. stem | 348 | 67 | 222 | 152 | 117 | 329 | 166 | 146 | 191 |
| Root vs. leaf | 407 | 78 | 255 | 181 | 145 | 391 | 197 | 171 | 232 |
| Root vs. rhizome | 406 | 66 | 258 | 162 | 127 | 379 | 182 | 157 | 218 |
| Root vs. stem | 383 | 63 | 245 | 161 | 129 | 364 | 172 | 152 | 212 |
| Stem vs. leaf | 240 | 39 | 152 | 98 | 75 | 225 | 105 | 89 | 124 |
