# Supplementary material for: Revealing the mechanisms of the bioactive ingredients accumulation in Polygonatum cyrtonema by multiomics analyses
Source: Front Plant Sci. 2022 Nov 16;13:1055721. doi: 10.3389/fpls.2022.1055721 (PMC9709641; doi:10.3389/fpls.2022.1055721)
Supplement: Supplementary file 3 [file Table_3.doc]

**Highlights**

The intermediates of flavonoids and saponins pathways were significantly up-regulated in the stem and leaf compared with rhizome.

*PcFK*, *PcF3H*, *PcAMY*, *PcCYP71A1*, and *PcSUS* exhibited a significantly high expression level and were associated with secondary metabolites accumulation*.*

*PcMYB3*, *PcMYB97*, *PcMYB102*, *PcMYB33*, and *PcMYB61* are correlated with flavonoids content.

A total of 169 miRNAs and 3,432 target genes were identified.

aof_miR164 was negatively correlated with *PcAOS*, *PcSPLA2*, *PcFRK1*, and *PcDELLA*.
